# Prescriptive Predictors of Mindfulness Ecological Momentary Intervention for Social Anxiety Disorder: Machine Learning Analysis of Randomized Controlled Trial Data

**DOI:** 10.2196/67210

**Published:** 2025-05-13

**Authors:** Nur Hani Zainal, Hui Han Tan, Ryan Yee Shiun Hong, Michelle Gayle Newman

**Affiliations:** 1 Department of Psychology National University of Singapore Singapore Singapore; 2 Department of Psychology The Pennsylvania State University University Park, PA United States

**Keywords:** digital mental health interventions, ecological momentary intervention, longitudinal, machine learning, mindfulness, precision medicine, randomized controlled trial, social anxiety disorder, digital mental health, intervention, ecological, anxiety, predictor, depression severity, mobile phone

## Abstract

**Background:**

Shame and stigma often prevent individuals with social anxiety disorder (SAD) from seeking and attending costly and time-intensive psychotherapies, highlighting the importance of brief, low-cost, and scalable treatments. Creating prescriptive outcome prediction models is thus crucial for identifying which clients with SAD might gain the most from a unique scalable treatment option. Nevertheless, widely used classical regression methods might not optimally capture complex nonlinear associations and interactions.

**Objective:**

Precision medicine approaches were thus harnessed to examine prescriptive predictors of optimization to a 14-day fully self-guided mindfulness ecological momentary intervention (MEMI) over a self-monitoring app (SM).

**Methods:**

This study involved 191 participants who had probable SAD. Participants were randomly assigned to MEMI (n=96) or SM (n=95). They completed self-reports of symptoms, risk factors, treatment, and sociodemographics at baseline, posttreatment, and 1-month follow-up (1MFU). Machine learning (ML) models with 17 predictors of optimization to MEMI over SM, defined as a higher probability of SAD remission from MEMI at posttreatment and 1MFU, were evaluated. The Social Phobia Diagnostic Questionnaire, structurally equivalent to the *Diagnostic and Statistical Manual* SAD criteria, was used to define remission. These ML models included random forest and support vector machines (radial basis function kernel) and 10-fold nested cross-validation that separated model training, minimal tuning in inner folds, and model testing in outer folds.

**Results:**

ML models outperformed logistic regression. The multivariable ML models using the 10 most important predictors achieved good performance, with the area under the receiver operating characteristic curve (AU-ROC) values ranging from .71 to .72 at posttreatment and 1MFU. These prerandomization and early-stage prescriptive predictors consistently identified which participants had the highest probability of optimization of MEMI over SM after 14 days and 6 weeks from baseline. Significant predictors included 4 strengths (higher trait mindfulness, lower SAD severity, presence of university education, no current psychotropic medication use), 2 weaknesses (higher generalized anxiety severity and clinician-diagnosed depression or anxiety disorder), and 1 sociodemographic variable (Chinese ethnicity). Emotion dysregulation and current psychotherapy predicted remission with inconsistent signs across time points.

**Conclusions:**

The AU-ROC values indicated moderately meaningful effect sizes in identifying prescriptive predictors within multivariable models for clients with SAD. Focusing on the identified notable client strengths, weaknesses, and Chinese ethnicity may enhance our ability to predict future responses to scalable treatments. Estimating the likelihood of SAD remission with a “prescriptive predictor calculator” for each client may help clinicians and policymakers allocate scarce treatment resources effectively. Clients with high remission probability may benefit from receiving the MEMI as a vigilant waitlist strategy before intensive therapist-led psychotherapy. These efforts may aid in creating actionable treatment selection tools to optimize care for clients with SAD in routine health care settings that use stratified care principles.

**Trial Registration:**

OSF Registries 10.17605/OSF.IO/M3KXZ; https://osf.io/m3kxz

## Introduction

Social anxiety disorder (SAD) is a global public health concern with high current prevalence rates ranging from 2% to 25% [1-4]. If left untreated, SAD could raise socioeconomic burdens by limiting educational and career attainment as well as impairing physical health [1,5]. Individuals struggling with SAD exhibit an enduring apprehension and avoidant habitual reactions to social or performance settings with fears and worries of negative social evaluation [6]. They also maintain fewer confidants, spend less time with friends, and persistently report negative moods [7], likely interfering with daily functioning [8]. Reviews consistently showed that heightened SAD was linked to negative self-views [9], frequent health care service usage [10], and worse quality of life [11]. Given its early onset and debilitating course [12], addressing the accessibility and scalability of treatment options for SAD is imperative.

Meta-analyses of randomized controlled trials (RCTs) evidenced the efficacy of labor-intensive mindfulness-based interventions (MBIs) for SAD [13]. Face-to-face 8- to 12-week MBIs, such as mindfulness-based stress reduction (MBSR [14]) and mindfulness-based cognitive therapy (MBCT [15]), dominate conventional delivery approaches [16]. These MBIs typically included intensive retreats for mindfulness meditation, lasting from 3 days to 3 months [17]. However, stigma, logistical, financial, and time constraints [18] hinder accessibility, highlighting the importance of digital, self-guided MBIs as a scalable solution for SAD.

Such digital and self-guided MBIs yielded small yet clinically meaningful decreases in social anxiety symptoms and subjective attention control deficits within 2-14 weeks [19,20]. Thus, even brief digital MBIs are efficacious at least in the short run. These brief self-guided MBIs can take a number of forms. For example, concise online interventions, consolidate core psychotherapy components in 1-6 hour-long sessions to encourage behavioral changes [21,22]. However, such online interventions are typically conducted within full sit-down sessions and there are questions about whether such hour-long weekly session interventions optimally generalize to individuals’ day-to-day lives and provide more long-term change. It is also difficult to know whether such sessions will generalize to the real world and day-to-day practice [23,24].

To address this concern, there has been more development of in-the-moment digital interventions provided and prompted in individuals’ day-to-day lives via smartphone apps (also known as ecological momentary interventions [EMIs] [21]). EMIs leverage smartphones using web-based platforms with varying time courses, with or without human coach support [25,26]. These EMIs consistently offer real-time therapy content to boost emotion regulation (ER) and target SAD-associated symptoms [27]. Mindfulness ecological momentary interventions (MEMIs) provide concise and real-time prompts to apply therapy skills at the moment and thus could be superior to conventional digital MBIs by seamlessly integrating practices into everyday routines, targeting daily stressors and symptoms, and strengthening new ER habits [28-31].

Nonetheless, there remains a paucity of knowledge of individual predictors of variable response to brief MEMIs for SAD. RCTs offer aggregate-level efficacy estimates, however, they do not reveal patient-level variations in treatment response. Even small between-treatment effect sizes may conceal heterogeneous treatment effects [32]. At the same time, systematic reviews (eg, [33]) and empirical studies (eg, [34] indicated that symptom and treatment variables might predict individual differences in MBI efficacy. Investigating treatment response variability, also called heterogeneous treatment effects [35], is critical for identifying which individuals with SAD gain the most from brief MEMIs.

Research on heterogeneous treatment effects in SAD has primarily focused on resource-intensive, face-to-face, nonscalable psychotherapies rather than scalable MBIs. Studies have tested patient attributes, symptom severity, and treatment processes as plausible predictors of CBT efficacy [36-40]. For instance, meta-regression showed that greater baseline symptom severity predicted better response to CBT for SAD [41], whereas delivery approach and treatment duration were nonsignificant moderators of intensive MBIs [42]. Expanding this investigation to brief MEMIs for SAD is crucial to better inform treatment-matching.

Precision medicine approaches may optimize treatment-matching by tailoring brief MEMIs to people with SAD based on their unique baseline attributes to improve patient-centered care [43]. Machine learning (ML), which prioritizes prediction and explanation [44], can model complex (eg, nonlinear), multivariable, high-dimensional interactions to identify prescriptive predictors (treatment efficacy moderators [45]). Unlike ordinary least squares (OLS) regression methods, ML approaches could better identify nonlinear associations and moderation effects, enhancing the prediction of heterogeneous treatment effects [46,47]. Moreover, the ability of ML to generalize accurate predictions to new, previously unseen data renders it well-suited to guide and optimize treatment selection [48]. Despite its potential, no studies have leveraged ML to predict heterogeneous treatment effects for brief MEMIs in SAD.

Integrating theories, such as capitalization versus compensation models [49], into ML methods can enhance variable selection and generalizability and minimize false discoveries by testing a prescriptive predictor set solely informed by theory, logic, and research [50]. The compensation model proposes that treatment response relies on effectively addressing patients’ disorder-specific vulnerabilities and deficits [49]. Conversely, the capitalization model posits that treatment response is likely greater when harnessing clients’ relative strengths [51].

Aligned with the compensation model, clients with SAD who display excessive perseverative cognitions, increased avoidance, greater depression severity, and impaired attention may benefit from MEMIs [49,52-56]. This is likely because MEMIs repeatedly teach nonjudgmental acceptance, present-mindedness, and valued activities. Consistent with the capitalization model, clients with fewer baseline SAD symptoms [57,58], higher self-compassion [59,60], stronger treatment credibility and expectancy [56], no current psychotropic medication, and greater trait mindfulness [13] benefitted from brief MBIs [56-61]. This may have been because these strengths helped increase engagement and positive self-fulfilling prophecies [51]. Findings on ER as a prescriptive predictor have been mixed [62,63], highlighting the importance of further research. Together, individuals with such profiles would likely benefit more from MEMIs for SAD than a self-monitoring-only app (SM).

Building on previous research, this study examined novel prescriptive predictors of a brief, fully self-guided, scalable MEMI for SAD. We extended an earlier RCT [64,65] on generalized anxiety disorder (GAD) to a new SAD sample [66]. This RCT showed that both MEMI and SM produced longer-term effects on SAD and its comorbid symptoms and risk factors, with significant between-group differences in momentary anxiety, depression, and mindfulness but not retrospectively reported symptoms [66]. Hypotheses were 2-fold. First, we predicted that multivariable prescriptive ML models would show acceptable performance (area under the receiver operating characteristic curve [AU-ROC] ≥.70) in predicting SAD remission (ie, absence of diagnosis based on the Social Phobia Diagnostic Questionnaire [SPDQ] [67]) at posttreatment and 1-month follow-up (1MFU; hypothesis 1). Acceptable model performance predicting differential efficacy to scalable interventions is crucial for building an actionable treatment selection tool. Second, we hypothesized that we could identify baseline variables that would predict the superiority of MEMI over SM at posttreatment and 1MFU SAD remission for individuals. In particular, we predicted that higher SAD, GAD, depression severity, perseverative cognitions, clinician-diagnosed anxiety or depression, as well as poorer trait mindfulness, and attention control, would predict better outcomes from MEMI (compensation model). We also predicted that higher compassion, treatment expectancy, credibility, absence of current psychotropic medication use, university education, and lower emotion dysregulation would predict better outcomes from MEMI versus SM (I model; hypothesis 2). Four other examined variables (age, sex, race, and current psychotherapy) were exploratory. Identifying prescriptive predictor patterns could help guide treatment-matching and optimize patient-centered care for SAD using under-investigated brief scalable MEMIs.

## Methods

### Participants

In our preregistered RCT [68,69], we used a 2 (treatment: MEMI, SM) by 3 (time: preintervention, posttreatment, 1MFU) mixed design to evaluate the efficacy of 14-day MEMI against SM in addressing clinical outcomes (Multimedia Appendix 1). Treatment assignment (MEMI or SM) was the between-person factor, whereas time was the within-person factor. This design for identifying treatment moderators with 17 prescriptive predictors using precision medicine was appropriate for several reasons. First, the assessor-blinded, balanced RCT design permits the adjustment of measured and unmeasured covariates and facilitates causal inference as part of developing the treatment selection tool [70]. Second, 2 armed RCTs facilitate identifying prescriptive predictors of the superiority of an active treatment to a control condition, which helps identify predictors specific to one form of treatment [71]. Also, the presence of randomization minimized selection bias, which could hinder the detection of prescriptive predictors [72]. Third, examining remission likelihood at 3-time points (pretreatment, posttreatment, and 1MFU) enabled testing whether prescriptive predictor patterns were generalizable across timeframes, offering more confidence in reproducible findings.

### Ethical Considerations

The current secondary analysis of an individually randomized parallel-group, assessor-blinded RCT received ethical approval from the National University of Singapore (NUS). All participants offered voluntary informed consent and could withdraw at any time without penalty. Deidentified data were collected and stored on a secure-encrypted cloud server. Participants were reimbursed monetarily or through course credits for their participation.

Eligible participants met the criteria of self-reported SAD, defined as a Social Phobia Inventory (SPIN) [73] score of ≥20. The ≥20 SPIN score discriminated individuals with and without SAD in previous research, balancing both sensitivity and specificity [73-75]. Eligibility also included being aged 18 or older, possessing a smartphone, and actively seeking assistance for mental health concerns. To enhance participant safety and eliminate those unlikely to benefit from the interventions, exclusion criteria encompassed those who self-reported suicidal thoughts, mania, or psychosis. We recruited individuals from the psychology subject pool and local community.

Participants (N=191) were randomized to two groups: 96 in MEMI and 95 in SM (Table 1). On average, they were 21.84 (SD 3.37, range 18-53) years old, with 21.47% (14/191) identifying as male, 78.01% (149/191) as female, and 0.52% (1/191) as other; 86.39% (165/191) identified as Chinese, 2.09% (4/191) as Malays, 6.28% (12/191) as Indian, and 5.24% (10/191) as other; 87.43% (167/191) were single, in contrast to those who were married, cohabiting, or in an intimate relationship but not residing together. The highest level of education achieved by 75.92% (145/191) of participants was junior college, as opposed to earning a diploma, university degree, or graduate degree. Psychotropic medications had been used by 5.24% (10/191) of individuals, and 15.71% (30/191) had received psychotherapy. For more information, refer to the Consolidated Standards of Reporting Trials (CONSORT)-eHEALTH checklist [76] in Multimedia Appendix 2.

**Table 1:** Sociodemographic characteristics of participants (N=191).

		Values
Age (years), mean (SD)		21.84 (3.37)
**Gender, n (%)**		
	Male	41 (21.47)
	Female	149 (78.01)
	Other	1 (0.52)
**Ethnicity, n (%)**		
	Chinese	165 (86.39)
	Malays	4 (2.09)
	Indians	12 (6.28)
	Others	10 (5.24)
**Marital status, n (%)**		
	Married with spouse	1 (0.52)
	Living with partner	1 (0.52)
	In an intimate relationship but not living together	22 (11.52)
	Never married	167 (87.43)
**Education, n (%)**		
	Junior college	145 (75.92)
	Diploma	12 (6.28)
	University degree	27 (14.14)
	Graduate degree	7 (3.66)
**Employment status, n (%)**		
	Full-time	12 (6.28)
	Part-time	40 (20.94)
	Not employed	139 (72.77)
**Student status, n (%)**		
	Full-time	178 (93.19)
	Part-time	5 (2.62)
	Not a student	8 (4.19)
**Annual salary** ^a^ **, n (%)**		
	$0-$10,000	172 (90.05)
	$10,001-$20,000	3 (1.57)
	$20,001-$40,000	7 (3.66)
	$40,001-$65,000	6 (3.14)
	$65,001-$100,000	3 (1.57)
**Psychotropic medication, n (%)**		
	No	181 (94.76)
	Yes	10 (5.24)

^a^This refers to Singapore dollars (SGD), with an exchange rate of 1 SGD to US $0.75 US at the time of the study.

### Self-Report Measures

#### Social Anxiety Disorder Severity

The 25-item SPDQ [67] assessed SAD fear and avoidance symptoms across different social situations per DSM-IV (*Diagnostic and Statistical Manual of Mental Disorders, Fourth Edition*) criteria. It has demonstrated good retest reliability and strong internal consistency (Cronbach α=0.96, 0.97, 0.98 at prerandomization, posttreatment, and 1MFU herein). The SPDQ evidenced strong discriminant validity, good convergent validity [67], and excellent sensitivity to change in RCTs [66].

#### Generalized Anxiety Disorder Severity

The 14-item generalized anxiety disorder Questionnaire-IV (GADQ-IV [77]) measured symptoms of GAD through a combination of binary (“Yes” or “No”) and continuous responses, including two 9-point Likert scales to assess interference and distress caused by GAD symptoms [77]. It exhibited strong internal consistency (α=0.93 at prerandomization) and robust retest reliability [77]. In addition, it exhibited strong convergent and discriminant validity and demonstrated good concordance with structured diagnostic evaluations of GAD [77,78].

#### Depression Severity

The 21-item Beck Depression Inventory-Second Edition (BDI-II [79]) assessed the severity of depression symptoms. Participants selected the severity level (ranging from 0-3) that best matched their experience with each symptom over the past 2 weeks. The BDI-II exhibited strong internal consistency (α=.93 at prerandomization) and strong convergent and discriminant validity [80].

#### Baseline Clinical Variables

Participants responded to these questions: “Are you currently diagnosed with a psychological disorder or condition? If yes, please indicate the disorder or condition.” “Have you ever been prescribed medications for emotional or psychiatric problems?” “Have you ever been in therapy or counseling?” and “What types of treatment are you currently receiving? You can choose multiple answers. (psychotherapy, medication, other [please specify], and not applicable).” The variables “current psychotherapy” and “psychotropic medication use” were derived from these questions and included in the predictor set. Data on changes in medication status were not collected at posttreatment and 1MFU.

#### Trait Emotion Dysregulation

The 36-item Difficulties in Emotion Regulation Scale (DERS [81]) measured participants’ emotion dysregulation, including emotional confusion, goal inertia, nonacceptance, self-awareness limitations, and skill deficits. Responses were recorded on a 5-point Likert scale, ranging from 1 (almost never) to 5 (almost always). Research indicated excellent internal consistency (α=.86 at prerandomization), acceptable retest reliability [82], good convergent, and strong discriminant validity [83].

#### Trait Self-Compassion

The 26-item Self-Compassion Scale (SCS) [84] assessed trait-level self-compassion, including dimensions of common humanity, isolation, overidentification, mindfulness, self-judgment, and self-kindness. Responses were captured on a 5-point Likert scale, ranging from 1 (almost never) to 5 (almost always). Internal consistency was high herein (α=.92 at prerandomization). SCS scores also showed good retest reliability [85], strong convergent validity, and excellent discriminant validity [86].

#### Trait Perseverative Cognitions

The 45-item Perseverative Cognitions Questionnaire (PCQ [87]) assessed persistent, perseverative cognition tendencies associated with obsessive, ruminative, and worrisome thoughts. Participants rated items using a 6-point Likert scale ranging from 0 (strongly disagree) to 5 (strongly agree). It included 6 domains: anticipating negative outcomes, dwelling on the past, preparing for the future, thoughts conflicting with the ideal self, perceived lack of control, and searching for causes and meanings. It showed good internal consistency (α=.96 at prerandomization), strong 2-week retest reliability, excellent discriminant, and convergent validity, and cross-cultural measurement equivalence between the United States and Singapore [88].

#### Trait Mindfulness

The 39-item Five-Facet Mindfulness Questionnaire (FFMQ [89]) evaluated participants’ inclination to practice mindfulness in 5 domains: observation, nonreactivity to inner experiences, nonjudgment, description, and awareness of the consequences of actions. It used a 5-point Likert scale, ranging from 1 (never or very rarely true) to 5 (very often or always true). The overall score of the FFMQ has shown strong convergent validity, differentiation from measures of unrelated constructs (eg, psychological well-being [89]), and good retest reliability. The α value was .90 at prerandomization.

#### Trait Attention Control

The Attentional Control Scale (ACS [90]) comprised 20 items, merging a 9-item measure of attention focusing with an 11-item measure of attention shifting. The ACS has good convergent and predictive validity [91], acceptable discriminant validity [92], and excellent retest reliability. The α value was .87 at prerandomization.

#### Treatment Credibility and Expectancy

Following the presentation of the video presentation of the therapeutic rationale in each group, participants completed the 6-item Credibility and Expectancy Questionnaire (CEQ [93]) to assess their belief in the credibility of the intervention and its potential to alleviate symptoms. Examples include “By the end of the therapy period, how much improvement in your symptoms do you think will occur?” and “By the end of the therapy period, how much improvement in your symptoms do you really feel will occur?” The CEQ demonstrated robust retest reliability and excellent internal consistency [93].

### Procedure

Initially, eligible participants completed a set of counterbalanced questionnaires using the counterbalancing functionality in Qualtrics. Counterbalancing minimizes sequencing influences by randomizing the presentation order of individuals completing unique self-assessments. This method prevented possible biases due to a fixed presentation order of the self-assessments, such as carry-over effects, fatigue, and practice effects [94]. Participants were then randomly assigned to MEMI or SM using the random generator function (RAND) of Microsoft Excel with permuted blocks of various sizes (2, 4, and 6) to create unpredictability [95]. This method gave every participant an equal probability of either group allocation. Allocation concealment was conducted by blinding research personnel who collected and analyzed data from the assigned group to maintain randomization integrity and prevent bias [96].

After completing all pretreatment assessments, the relevant group-assigned video using Qualtrics was provided toward the conclusion of the baseline visit. Participants installed the Personal Analytics Companion (PACO) app [97], which housed either MEMI or SM on their smartphones, with the video demonstrating its features. Participants were informed that they would receive prompts at 5 daily intervals (around 9 AM, noon, 3 PM, 6 PM, and 9 PM) during the subsequent 14-day period. Prompts could be adjusted to suit participants’ schedules. To maintain validity, participants were instructed to input their responses on state depression, anxiety, and mindfulness both before and after the MEMI or SM induction within a 2-hour window of the prompt. Based on their assigned group, the app prompted guided participants to continually practice mindfulness or self-observation skills.

### Group Characteristics

#### Mindfulness Ecological Momentary Intervention

Participants received a standardized video presentation in which the principal investigator explained evidence-based MBI protocols similar to MBSR. The video guided MEMI participants to immerse themselves in the present moment and empowered them with open monitoring and attentive engagement skills (ie, to attend to temporary moments). Afterward, the video therapist showcased the skill of paced, rhythmic diaphragmatic breathing and its application in practice. Diaphragmatic breathing retraining, resembling mindful breath awareness, has demonstrated effectiveness in both clinical [14,98] and nonclinical settings [99,100]. The video therapist continued by teaching nonjudgmental acceptance, incorporating elements of MBCT such as diaphragmatic breathing and mindfulness practices such as observation, nonreactivity, and nonjudgmental acceptance. These therapy components were selected to reduce SAD symptoms and self-criticism [13] and enhance ER and self-compassion [101]. Diaphragmatic breathing could induce physiological changes, such as lowering resting heart rate in conjunction with anxiety symptoms [102]. Next, each MEMI participant was informed about the importance of consistent mindfulness practice. The 6-item CEQ was then administered, and participants set up the MEMI on their smartphones (Multimedia Appendix 3). They were also provided with the MEMI treatment rationale handout via Qualtrics and encouraged to engage with mindfulness skills consistently.

#### Self-Monitoring

In the SM video presentation, the principal investigator began by defining self-observation as a heightened awareness of one’s thoughts and emotions, with a particular emphasis on distressing experiences. The video suggested that monitoring thoughts and observing associated distress alone could encourage a healthier mindset. Essentially, the SM video conveyed that focusing solely on distress had the potential to alleviate anxiety symptoms. A recent and brief app intervention explanation inspired the rationale for SM as a placebo control [103-105]. Our SM adaptation sought to mimic MEMI while excluding its theorized active therapeutic elements, including acceptance, breath retraining, open monitoring, mindfulness of temporary moments, and regular mindfulness practice. It refrained from referencing mindfulness, excluded directions to heighten awareness of current experiences adaptively, and avoided guiding participants toward mood-altering engagement with the present moment. In contrast to MEMI, SM encouraged participants to observe their thoughts and emotions without instructing them to accept those thoughts and feelings as they arose. Breathing retraining guidance was missing, and there was no instruction to elicit calming sensations linked to diaphragmatic breathing. Whereas MEMI placed importance on developing skills persistently, SM participants were not urged to engage in self-observation between prompts or after the 14-day treatment phase (Multimedia Appendix 4). SM was designed to adjust for potential credibility, expectancy, and placebo effects, minimize regression to the mean, and decrease the likelihood of inflated effect sizes that might occur with waitlists or no-treatment controls [106,107]. Further details on treatment engagement, protocol fidelity, and rationale are provided in Multimedia Appendix 1.

### Data Analyses

Our study adhered to the Transparent Reporting of Multivariable Prediction Model for Individual Prognosis or Diagnosis (TRIPOD) guidelines for conducting and reporting our research [108,109]. Missing data (10% [344/3438] in each training and test fold) was managed using random forest (RF) imputation [110] with the missRanger R package (R Core Team [111]) embedded in the nestedcv R package [112]. RF imputation was conducted instead of multiple imputations as it better handles diverse forms of missing data (eg, nonparametric relations), nonlinearities, and higher-order interactions, and generates less biased and narrower uncertainty estimates [113]. Model performance metrics also improved with RF imputation compared to multiple imputations in building multivariate ML models [113,114]. Imputation and standardization were not conducted in the whole dataset, as doing so would result in information leakage [115]. Thus, imputation was conducted separately on the training fold in the inner cross-validation loop and the test folds in the outer cross-validation loops of the ML analysis detailed below. Continuous variables were normalized to mean of 0 and SD of 1, and nominal variables were one-hot encoded separately in the training and test folds [112].

A simulation-based sample size determination method [116-118] indicated that to detect 2-way interactions of treatment x predictor for a binary remission outcome, a sample size of at least 150 was required with 17 initial predictors. The minimum sample size needed with the final top 10 prescriptive predictors was 100. Multimedia Appendix 1 details this sample size determination method in the context of multivariable ML analyses.

To explore potentially prescriptive predictive models, we employed two algorithms—RF and support vector machine (SVM)—that are appropriate for relatively small sample sizes. RF trains a parallel ensemble of decision trees by drawing random samples from a dataset and including stop rules. Decision trees use training data to create a tree structure that forms branches at each predictor, enabling the prediction of outcomes. RF offers advantages such as decreased error rate, reduced susceptibility to overfitting (generalizability issues), and diminished influence of outliers over decision trees [119]. RFs can effectively manage correlations among predictors by automatically decorrelating decision trees. In addition, SVM classifies data using a hyperplane that optimizes the separation distance between groups, relying on input predictors. SVM outperforms logistic regression when groups are easily separable on outcomes of interest [120]. Both algorithms excel at handling sparse datasets with sizeable predictor-to-sample size ratios [121-124]. Also, the logistic regression algorithm used maximum likelihood estimation and logistic link function as a reference model. The AUCs of RF and SVM over logistic regression were compared using DeLong’s test [125,126].

Instead of relying on a conventional training-test set or nonnested k-fold cross-validation approaches, we used nested 10-fold cross-validation (10F-CV) with 10 repetitions and grid-search [127]. Nested 10F-CV provides more robust performance estimates for small sample sizes, maximizes data utilization, minimizes bias, and controls for overfitting more than the split train-test approach [128,129]. It subsets the dataset into 10 outer and 10 inner folds [130]. Inner cross-validation is used to refine model selection and minimal tuning. In the outer folds, 10% (344/3438) of the data was reserved for model testing, whereas the remaining data was used for model development. This process was reiterated 10 times, each selecting a distinct 10% (344/3438) of the data for validation with unseen test data while training a new model using the remaining 90% (3094/3438). The overall performance was determined by calculating the mean classification performance of 10 independently developed models on distinct 10% (344/3438) subsets of the validation data not used in the model development process ([131]; refer to Multimedia Appendix 5, which elaborates on the rationale and procedures of these ML methods).

Using a counterfactual causal inference approach, we harnessed a 2-model learner (T-learner) approach to estimate remission probabilities for each individual across treatment arms, regardless of their actual intervention assignment (refer to 35 for a step-by-step tutorial and Multimedia Appendix 4 for a summary of the assumptions for this approach). This method entailed training RF models separately for the treated (MEMI) and control (SM) arms to predict remission rates within each arm [132]. We then applied these models to impute the predicted remission probabilities for each individual as though they had received each treatment, irrespective of their actual assignment. To improve the accuracy of these predictions and reduce overfitting, we used nested 10F-CV to maximize the likelihood of model estimates staying generalizable and robust [130]. The present method provided a nuanced comprehension of heterogenous treatment effects by generating participant-level predicted (or imputed) remission probability estimates (ranging from 0% to 100%) under both scenarios—treated and untreated—thus demonstrating each treatment’s possible advantages and shortcomings for each person [133]. Participants were regarded as optimized to MEMI if their imputed remission probability scores were higher for MEMI than SM. Heterogeneous treatment effects were then identified by computing the difference in imputed remission probabilities between the two treatments for each individual. This difference score was used to determine which treatment generated a greater remission probability for each participant [134]. Participants were regarded as optimized to MEMI if their imputed remission probability scores were higher for MEMI than SM. This approach empowers researchers to efficiently identify subgroups with greater odds of gaining from particular treatment options, potentially offering personalized interventions.

Remission was defined as posttreatment and 1MFU SPDQ scores <12.13, as this cut-off yielded high specificity (true negative cases accurately detected; 134/143, 94%) and good classification accuracy (correct categorization into respective classes; 100/143, 70%) with SAD diagnosis [67]. For each individual, optimization to MEMI was coded as “1” versus “0” if the remission likelihood with MEMI exceeded the remission likelihood with SM. The initial ML model included 17 baseline predictors: age, gender, ethnicity, education, treatment credibility, expectancy, SAD, depression, GAD severity, clinician-diagnosed depression or anxiety disorder, baseline psychotherapy, current psychotropic medication use, trait attentional control, emotion dysregulation, mindfulness, perseverative cognition, and self-compassion. The final model comprised 10 top predictors of optimization to MEMI, which were selected through the elastic net regression algorithm variable screening filter and were well-suited for nested CV ML analyses [112].

For several reasons, the predictor set did not include intermediate variables not obtained at baseline, such as intervention engagement. First, including postbaseline variables would compromise randomization benefits, thereby biasing estimates of differential efficacy [135]. Second, adding postbaseline, intermediate variables into the prescriptive predictor models could lead to optimism bias, indicated by inflated effect sizes [136,137]. Third and most importantly, including variables, such as engagement, that can only be obtained later during the trial prevents building a clinically actionable and scalable multivariable model that could inform treatment selection from the outset solely using accessible baseline data [138].

Similar to the above, the future change status of variables (eg, current psychotherapy and medication use) was excluded from the multivariate equation. Controlling for covariates that change at future time points, sometimes called controlling for postintervention factors, might result in biased estimates by inducing spurious relationships between the treatment and unmeasured predictors [139]. In prospective studies such as RCTs, variables assessed postintervention might function as colliders or mediators, and statistically controlling for them could create selection bias or obstruct the causal pathway [140,141]. Moreover, including change status covariates could result in overcontrolling, possibly generating spurious relationships or obscuring the estimation of true treatment effects and detection of prescriptive predictors [142].

AUC was used to assess classification performance. When AUC equals .50, it signifies that group differentiation was no more accurate than chance, whereas a range of .70-.79 indicates satisfactory differentiation, and an AUC of .80 or higher marks excellent differentiation. These guidelines also extended to two additional performance benchmarks: accuracy, which measures the proportion of correctly classified cases, and balanced accuracy (BAC), which represents the average of sensitivity (capacity to identify cases) and specificity.

To manage class imbalance in remission outcome, we applied the synthetic minority oversampling technique (SMOTE) by decreasing the overrepresented category while augmenting the underrepresented category to generate a more equitable dataset distribution [143]. SMOTE was implemented independently on the inner and outer folds to avoid data leakage and retain model evaluation integrity [144]. This procedure ensured that synthetic samples were created only within the training data of each fold, preventing contamination of the test set with artificially generated cases [145]. By performing SMOTE within each fold, model performance was more accurately tested with future unseen data, offering a robust estimate of its generalization abilities. This approach aligned with recommended practices in ML to manage imbalanced datasets while ensuring the reliability of CV outcomes [146].

Additional metrics were examined to test ML model performance more holistically. Sensitivity (true positive rate, which is also called recall) and specificity (true negative rate) were computed to evaluate the model’s capacity to accurately determine optimal versus nonoptimal cases, respectively [147]. Positive predictive value (PPV), also called precision, indicated the percentage of optimization to MEMI versus SM cases that were accurately predicted [148]. The *F*_1_-score, which balances precision and recall, offered a unique performance metric that considered false positives and negatives [149]. The area under the precision-recall curve (AUPRC) was computed to examine model performance under various thresholds, which could be particularly valuable for datasets with imbalanced outcome proportions [150]. Calibration analysis examined the degree to which predicted probabilities of optimization to MEMI aligned with actual outcomes (Multimedia Appendix 5), signifying the model’s reliability for clinical decision-making [151].

Although interpreting individual ML model coefficients can be risky due to their focus on overall prediction accuracy over individual coefficient precision, gaining insight into the direction of associations between predictors and outcomes remains valuable. We explored the significance and direction of predictors of optimization to MEMI by using the kernel Shapley additive explanations (SHAP) technique, using the fastshap R package [152]. SHAP is a versatile method for assessing the significance of predictors and the direction of predictor-outcome associations in multivariate ML prediction models across various contexts. All analyses were derived from published ML tutorials [153-155].

## Results

### Overview

**Figure 1:**
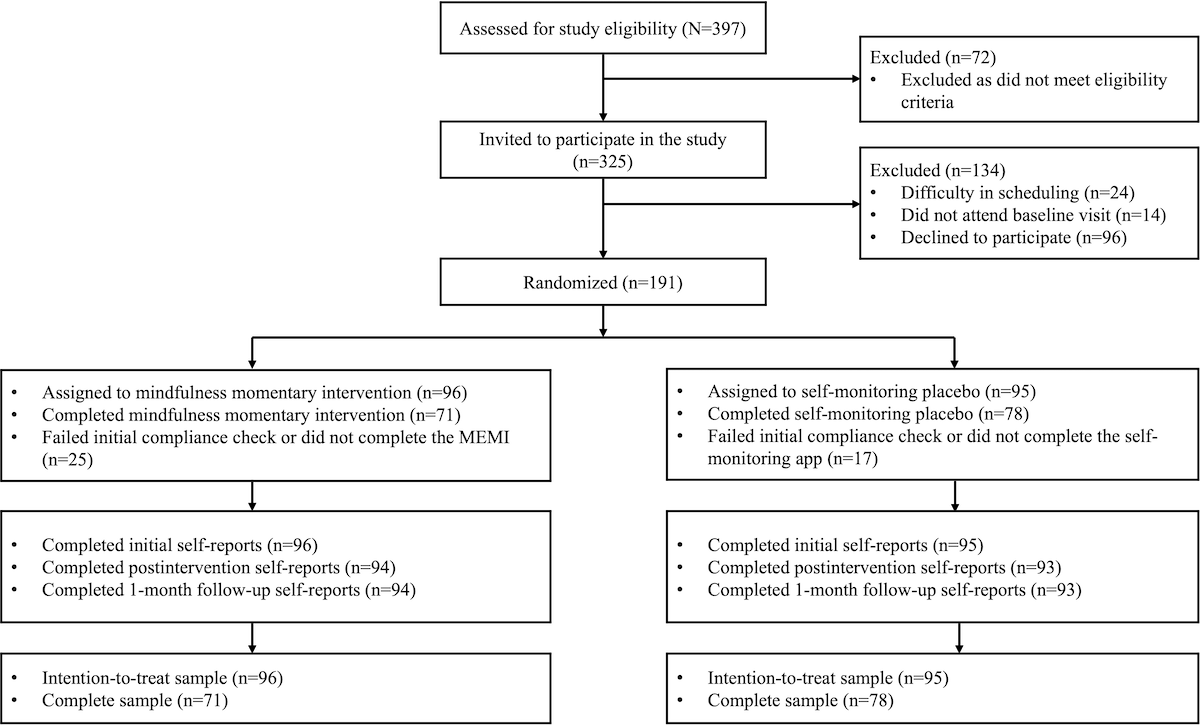
Consolidated Standards of Reporting Trials (CONSORT) diagram of recruitment flow.

Figure 1 illustrates the participant flow and recruitment process, following the Journal Article Reporting Standards [69].

### Engagement Rates Across Intervention Arms

Engagement rates were defined as the proportion of MEMI or SM prompts (out of 70) completed during the 14-day intervention phase. For MEMI participants, engagement was measured based on their completion of questions asked of them before and after each 1-2-minute prompt to engage in slow, rhythmic, diaphragm breathing, present-mindedness, acceptance, and attending to small moments while doing an activity (Multimedia Appendix 3). For SM participants, engagement was measured based on responses to the same questions after noticing their thoughts and feelings for about 30 seconds and how distressing they might be (Multimedia Appendix 4). Participants indicated that they completed instructions in either arm by selecting “Okay” to those instructions and completing the postprompt or induction items. An earlier primary efficacy study reported the manipulation check, showing evidence of the validity of the momentary induction of MEMI versus SM in the same sample [66]. By this definition, engagement was 85.3% (60/70; SD 16.8%, 12/70) in MEMI and 85.1% (60/70; SD 18.7%, 13/70) in SM. No significant between-group differences in engagement rates were observed (t_186.52_=–0.06, *P*=.95).

### Evaluation of Efficacy and Effectiveness on SPDQ Remission

From pre-post treatment, no significant between-group effects on SPDQ remission were observed (MEMI: 15.6%; SM: 21.1%; *χ*^2^_1_=0.94, *P*=.33). Likewise, no significant between-group effects on SPDQ remission occurred from pre-1MFU (MEMI: 26.0%; SM: 26.3%; *χ*^2^_1_=0.002, *P*=.97). Nonetheless, within-group remission rates in both groups significantly increased from pre-post treatment (MEMI: 0 to 15.6%; *χ*^2^_1_=16.27, *P*<.001; SM: 0 to 21.1%; *χ*^2^_1_=22.35, *P*<.001) and pre-1MFU (MEMI: 0 to 26.0%; *χ*^2^_1_=28.74, *P*<.001; SM: 0 to 26.3%; *χ*^2^_1_=28.79, *P*<.001).

### Testing Hypothesis 1 (Acceptable ML Model Performance)

#### Pre-Post Treatment Period

SVM was the best-performing initial ML model with all 17 baseline predictors (AUC=.70, 95% CI .68-.72, accuracy=.65, BAC=.65; refer to Table 2 for other performance metrics). Supporting hypothesis 1, the final SVM model with the top 10 predictors also yielded good performance (AUC=.71, 95% CI .69-.73, accuracy=.66, BAC=.66, AUPRC=.39). The AUC of the SVM (DeLong difference test *P*=.18) and RF model (DeLong difference test *P*=.23) were nonsignificantly better than the logistic regression model. Regarding calibration, the model-specific correlation between predicted probabilities and actual outcomes had a small-to-medium effect size (*d*=0.38; Multimedia Appendix 5).

**Table 2:** Model performance of the nested 10-fold cross-validation machine learning models predicting Social Phobia Diagnostic Questionnaire (SPDQ) remission.

Model			AUC^a^		LCI^b^		UCI^c^		Accuracy		BAC^d^		Sensitivity		Specificity		PPV^e^		*F*_1_-score		AUPRC^f^
**Logistic regression**																					
	Pre-post	.69		.68		.72		.65		.64		.63		.68		.66		.65		.39	
	Pre-1MFU^g^	.82		.82		.85		.75		.74		.69		.83		.80		.74		.34	
**Random forest**																					
	Pre-post	.69		.68		.72		.65		.65		.66		.68		.67		.67		.39	
	Pre-1MFU	.82		.82		.85		.75		.75		.70		.83		.80		.74		.34	
**Support vector machine**																					
	Pre-post (initial)	.70		.68		.72		.68		.72		.65		.68		.67		.66		.39	
	Pre-post (final)	.71		.69		.73		.69		.73		.66		.69		.68		.67		.39	
	Pre-1MFU (initial)	.83		.82		.85		.82		.85		.68		.82		.79		.73		.34	
	Pre-1MFU (final)	.72		.70		.73		.70		.73		.65		.71		.69		.67		.38	

^a^AUC: area under the receiver operating characteristic curve.

^b^LCI: lower bound of the 95% CI.

^c^UCI: upper bound of the 95% CI.

^d^BAC: balanced accuracy.

^e^PPV: positive predictive value.

^f^AUPRC: area under the precision-recall curve.

^g^1MFU: 1-month follow-up.

#### Pre-1MFU Period

SVM was the best-performing initial ML model with all 17 baseline predictors (AUC=.83, 95% CI .82-.85; accuracy=.74, BAC=.73; refer to Table 2 for other performance metrics). The AUC of the SVM (DeLong difference test *P*=.016) and RF (DeLong difference test *P*<.001) models were significantly better than the logistic regression model. Supporting hypothesis 1, the final SVM model with the top 10 predictors also generated good performance (AUC=.72, 95% CI .70-.73; accuracy=.68, BAC=.68, AUPRC=.38). Concerning calibration, the model-specific association between the predicted probabilities and actual outcomes had a small-to-medium effect size (*d*=0.37; Multimedia Appendix 5).

### Testing Hypothesis 2 (Theory-Driven Prescriptive Predictors)

#### Pre-Post Treatment Period

**Figure 2:**
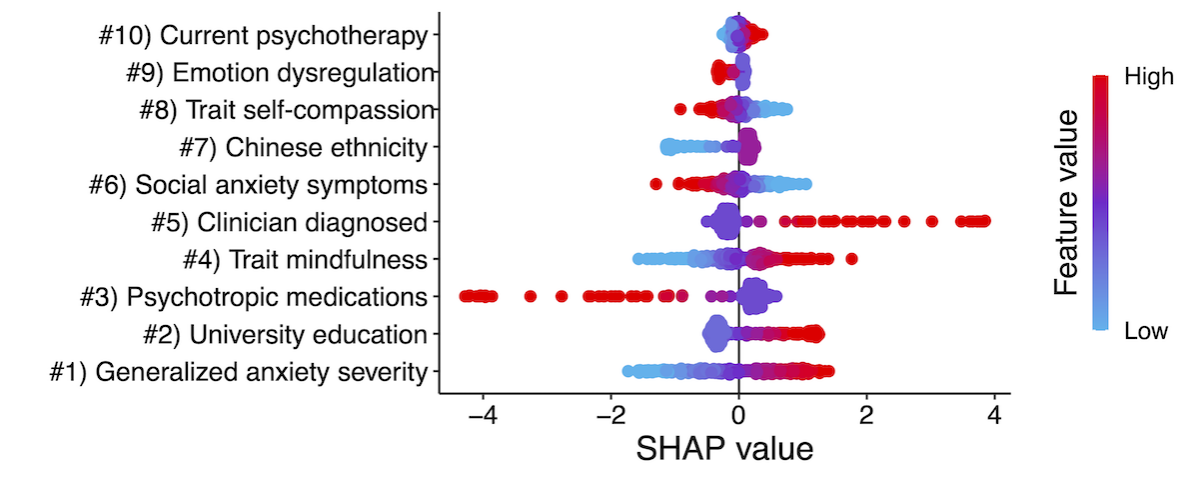
Top 10 prescriptive predictors of optimization to mindfulness ecological momentary intervention (MEMI) over self-monitoring app (SM) for social anxiety disorder at post-treatment in the final multivariate machine learning (ML) model. SHAP: Shapley additive explanations.

Figure 2 presents the directions of association between each predictor and probability of posttreatment SPDQ remission in the final multivariate model. Regarding strengths, higher trait mindfulness (4), lower SAD severity (6), absence of current psychotropic medication use (3), university education (2), and current psychotherapy (10) predicted optimization to MEMI over SM. With regard to weaknesses, higher GAD severity (1), clinician-diagnosed depression or anxiety disorder (5), lower trait self-compassion (8), and trait emotion regulation (9) predicted better outcomes from MEMI against SM. With respect to sociodemographic variables, ethnically Chinese (7) participants were more likely to experience optimization to the MEMI than SM. These outcomes were partially consistent with hypothesis 2.

#### Pre-1MFU Period

**Figure 3:**
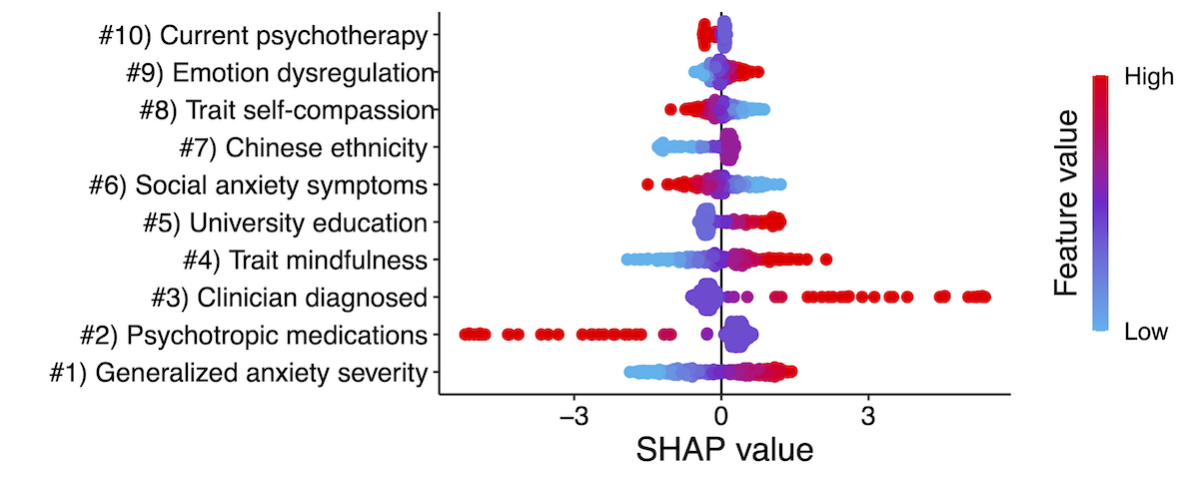
Top 10 prescriptive predictors of optimization to mindfulness ecological momentary intervention (MEMI) over self-monitoring app (SM) for social anxiety disorder at one-month follow-up (1MFU) in the final multivariate machine learning (ML) model. SHAP: Shapley additive explanations.

Figure 3 presents the directions of associations between each predictor and the probability of 1MFU SPDQ remission in the final multivariate model. Regarding strengths, higher trait mindfulness (4), trait emotion regulation (9), lower SAD severity (6), absence of current psychotropic medication use (2), university education (5), and current psychotherapy (10) predicted optimization to MEMI over SM. With regard to weaknesses, higher GAD severity (1), lower self-compassion (8), and presence of clinician-diagnosed depression or anxiety disorder (3) predicted optimization to MEMI. Ethnically Chinese (7) participants also had a higher probability of experiencing optimization to the MEMI than SM. These results were partially concordant with hypothesis 2. Consistent with this hypothesis, GAD severity, clinician-diagnosed anxiety or depression (cf compensation model), no psychotropic medication, and higher education (cf capitalization model) predicted optimization to MEMI. At the same time, findings that lower SAD severity, self-compassion, and higher trait mindfulness were prescriptive predictors of better outcomes from MEMI were also consistent with predictions.

## Discussion

### Principal Findings

We aimed to test the value of ML in predicting posttreatment and follow-up SAD remission after a course of brief MEMI compared to SM. Consistent with hypothesis 1, all multivariate ML models had AUC values at or above .70, indicating clinically meaningful, moderate effect sizes (refer to AUC to Cohen *d* effect size conversions by [156]). PPV or precision (percentage of participants accurately predicted to respond better to MEMI than SM) and other key metrics (sensitivity, specificity, and *F*_1_-score) also suggested acceptable model performance. SVM and RF notably performed better than logistic regression, concurring with previous precision psychiatry research [157] and indicating that these algorithms could outperform OLS regression to handle high-dimensional data sets to identify prescriptive predictors. Together, ML might hold promise in its ability to predict psychotherapy endpoints, including brief, fully self-guided smartphone apps, and pinpoint factors predicting success early in the process.

Offering partial concordance with our second hypothesis, 8 of 10 baseline variables consistently predicted optimization to MEMI over SM regarding SAD remission at posttreatment and follow-up: 4 strengths (lower SAD severity, higher trait mindfulness, absence of psychotropic medications, university education), 3 weaknesses (higher GAD severity, lower trait self-compassion, clinician diagnosis of anxiety and depression), and 1 demographic variable (Chinese ethnicity). Relatedly, 2 variables (current psychotherapy and trait emotion dysregulation) in the final multivariate predictive models showed inconsistencies in the direction or sign of predictor-outcome relations, signaling variable instability [158]. Further research is thus required to evaluate their potential importance with larger sample sizes.

Plausibly, clinicians should consider incorporating a focused set of relevant baseline data when using machine learning (ML) models to guide treatment decisions. This approach can help improve the accuracy of predictions while remaining practical for implementation in clinical settings [159,160]. Potential accounts are put forth in interpreting these 8 variables that consistently predicted optimization to brief, fully self-guided MEMI over SM. Relatedly, these findings should be interpreted in the context that each predictor adjusted for all other predictors in the final multivariate model.

Why did those with baseline lower SAD severity and stronger trait mindfulness respond better to MEMI than SM? Replicating prior work [57], higher SAD severity, which indicated persistent avoidance, precluded benefitting from low-intensity, brief, fully self-guided MEMI as there were no directives to actively expose themselves to various feared social situations [161]. Further, similar to a previous meta-regression of MBI RCTs for SAD [13], perhaps those with high-trait mindfulness were already good at exercising nonjudgmental acceptance of and nonreactivity to inner experiences. Thus, MEMI’s ongoing prompting may have further strengthened the habitual use of these skills (cf capitalization model [162]). This interpretation is aligned with a previous RCT, which showed that college students with high trait mindfulness were more likely to experience anxiety reductions after a brief MBI [163].

Two other noteworthy strengths that predicted optimization to MEMI against SM were not using psychotropic medications and university education status. Plausibly, patients taking psychotropic medications were less inclined to sustain improvements because they credited the gains to medication and thus ceased practicing mindfulness at follow-up. These interpretations aligned with the established depression literature [164-166]. Also, although the moderating role of education on EMIs has been mixed [167], higher education might correspond with stronger receptivity towards the MEMI.

Replicating and extending previous research [55,56], two baseline weaknesses—higher GAD severity and lower trait self-compassion—predicted better responses to MEMI against SM. Such findings might be attributed to evidence that brief 14-day MBIs could alleviate postevent brooding over social events. MEMI was most appropriate for high worriers, probably because its instructions of being present-minded, nonjudgmental, and accepting was the antithesis of worrying about the future (cf compensation model [49,64]). The outcome that lower trait self-compassion predicted higher benefits from MEMI might be because it was shown to differentially improve self-compassion domains for people with SAD, including self-kindness, interpersonal connectedness, and nonidentification with feelings [168].

Simultaneously, having been diagnosed with depression or anxiety disorders by a clinician predicted higher SAD remission probability via the MEMI over SM. Possibly, receiving an official diagnosis from a mental health care professional could boost their motivation to uphold their therapy skills practices. Future research could test the validity of these ideas by directly administering measures for the usage of therapy skills.

Also, those from the majority Chinese cultural group in Singapore (a Southeast Asian country), where our RCT was conducted, benefitted more from MEMI than SM compared to ethnic minority participants. These findings extended previous trials, which showed that MBIs, including the brief MEMI [168], produced stronger efficacy for White (vs non-White) individuals in mostly United States settings [169,170]. Since ethnic minorities comprised a disproportionately small proportion in this study (27/191, 14%), sampling error might have skewed outcomes toward the ethnic majority. Future studies should determine if a more balanced sample in terms of ethnic or racial composition might yield similar findings. Alternatively, modifying MEMIs for specific cultures might enhance their ability to meet the needs of ethnic minorities, which remain understudied in Asia [171]. However, the modifications rooted in real-life encounters (eg, institutional racism) demand thorough assessment due to the intricate interplay of intersectional factors (eg, economic and educational disparities) and varying conclusions regarding the efficacy of culturally tailored psychotherapies.

Seven variables were nonsignificant predictors: age, gender, treatment credibility, expectancy, self-reported depression symptom severity, trait attentional control, and repetitive thinking. Perhaps SAD patients with all levels of these baseline variables could benefit from the MEMI. The nonsignificance of age, gender, and trait repetitive thinking might suggest that those variables were not predictive of differential efficacy in the context of MEMI versus SM for SAD.

Findings should be interpreted considering study limitations. First, our models require further replication to determine their relevance across diverse samples. Enlarging the participant pool may enhance the accuracy of EMIs for SAD by using ML methods and customizing treatments based on individual traits. Nevertheless, ML-based multivariate predictive models using SVM and RF algorithms within nested cross-validation techniques allow for the development of treatment prediction models using modest sample sizes, such as ours, effectively tackling overfitting and class imbalance challenges [121,172,173]. Second, future endeavors should investigate the reproducibility of our ML models in the absence of SMOTE. Third, future research should expand the range of potential predictors to identify distinctive factors influencing differential intervention outcomes. Fourth, prudence should be exercised when inferring causality while interpreting the predictor outcome associations and rank variable importance [174]. Fifth, future studies could enhance calibration by employing more advanced ML approaches, such as deep learning or ensemble methods, and include more relevant predictors to improve discriminative power indexed by the AUPRC [175,176]. Sixth, the value of ML, such as RF, over traditional logistic regression merits ongoing investigation [126]. Seventh, ML models should serve as treatment planning heuristics rather than offering rigid directives. Eighth, the primarily Chinese (164/191, 83.39%) and female (149/191, 78.01%) demographic composition might limit the generalizability of results to other gender and racial groups. Ninth, as depending solely on self-report could be vulnerable to response fatigue, response shift, recall bias, and method variance [177], future similar studies should administer performance tasks (eg, behavioral avoidance tests) and biomarkers (eg, physiology [178]) to ensure successful extinction of fear. Tenth, changes in psychotropic medication status were not assessed at posttreatment and 1MFU. Future studies should gather such data and ensure stable medication doses in all participants to rule out the possibility that any observed efficacy was due to medication changes. Finally, the exclusion criteria encompassed those who self-reported suicidal thoughts, mania, or psychosis, thereby limiting the generalizability of results to SAD participants with these psychiatric comorbidities [25,72]. However, excluding these participants is a sound approach to preserving internal validity and maintaining participant safety. It concurs with ethical principles for research with vulnerable populations and aids with adjusting for potential confounds that might bias findings.

Limitations notwithstanding, study strengths included the execution of an RCT in Asia, an understudied region [88,179], and low attrition of 22% (42/191), which was lower than meta-analytic weighted attrition rates of mindfulness apps ranging from 24.7% (2287/9258) to 38.7% (3583/9258) [180]. Further, 78% (149/191) actively participated in at least 80% (56/70) of the EMI prompts. Another strength of the study was that some of the predictors examined (eg, current medication use, psychotherapy status) were unlikely to change during the 6-week study period. Finally, all the assessment tools used herein had a well-established history of use in RCTs and have shown good sensitivity to change [66,181-183].

### Conclusion

To conclude, the AUC values of .71-.72 of the prescriptive predictor models implied moderate performance, suggesting that more fine-tuning and validation are needed to raise confidence about their real-world clinical utility [184]. However, these findings offer an enhanced understanding of plausible prescriptive predictors of scalable MEMI outcomes for SAD. Broadly, clinical psychology encounters obstacles when implementing precision medicine approaches [185]. However, the present study adds to growing evidence that building ML-derived intervention allocation rules in examining which client with SAD benefits from the MEMI might enhance the caliber of data-driven clinical judgment. Thus the study provides potential precision treatment guidelines by means of uncertainty and probability framing [186] and prescriptive calculators [187]. Such prescriptive calculators should include significant predictors (in the present context, the 8 consistent predictors), exclude nonsignificant predictors (eg, age, gender), and focus on strengths and weaknesses, as informed by the capitalization and compensation models. Tailored treatments could enhance patient acceptance while optimizing the integration of evidence-based intensive psychotherapies in clinical services. Ultimately, though further refinement will be required to enhance prescriptive, predictive performance, incorporating EMI prescriptive models into routine care may enhance the effectiveness and efficiency of treating SAD within stratified care models [48].

## Appendix

Multimedia Appendix 1 Sample size determination and study procedure details.

Multimedia Appendix 2 CONSORT-eHEALTH checklist (V 1.6.1).

Multimedia Appendix 3 Screenshots for mindfulness ecological momentary intervention (MEMI) arm.

Multimedia Appendix 4 Screenshots for self-mointoring app (SM) arm.

Multimedia Appendix 5 Model assumptions and calibration plots.

## Abbreviations

10F-CV: 10-fold cross-validation
1MFU: 1-month follow-up
ACS: Attentional Control Scale
AUPRC: area under the precision-recall curve
AU-ROC: area under the receiver operating characteristic curve
BAC: balanced accuracy
BDI-II: Beck Depression Inventory-Second Edition
CEQ: Credibility and Expectancy Questionnaire
CONSORT: Consolidated Standards of Reporting Trails
DERS: Difficulties in Emotion Regulation Scale
DSM: Diagnostic and Statistical Manual
DSM-IV: Diagnostic and Statistical Manual of Mental Disorders, Fourth Edition
EMIs: ecological momentary interventions
ER: emotion regulation
FFMQ: Five-Facet Mindfulness Questionnaire
GAD: generalized anxiety disorder
GADQ-IV: Generalized Anxiety Disorder Questionnaire-IV
MBIs: mindfulness-based interventions
MBSR: mindfulness-based stress reduction
MEMI: mindfulness ecological momentary intervention
ML: Machine learning
NUS: National University of Singapore
OLS: ordinary least squares
PACO: Personal Analytics Companion
PCQ: Perseverative Cognitions Questionnaire
PPV: Positive predictive value
RAND: random generator function
RCTs: randomized controlled trials
RF: random forest
SAD: social anxiety disorder
SCS: Self-Compassion Scale
SHAP: Shapley additive explanations
SM: self-monitoring
SMOTE: synthetic minority oversampling technique
SPDQ: Social Phobia Diagnostic Questionnaire
SPIN: Social Phobia Inventory
SVM: support vector machine
TRIPOD: Transparent Reporting of Multivariable Prediction Model for Individual Prognosis or Diagnosis
