# Supplementary material for: Prescriptive Predictors of Mindfulness Ecological Momentary Intervention for Social Anxiety Disorder: Machine Learning Analysis of Randomized Controlled Trial Data
Source: JMIR Ment Health. 2025 May 13;12:e67210. doi: 10.2196/67210 (PMC12117280; doi:10.2196/67210)
Supplement: Multimedia Appendix 1 [file mental_v12i1e67210_app1.docx]

**Online Supplemental Materials**

**Multimedia Appendix 1**

**Sample size determination**

To determine sample size requirements for the present multivariable machine learning (ML) analysis, we developed an analytic script that uses a simulation-based method to compute the necessary sample size to attain an area under the receiver operating characteristic curve (AUC) in logistic regression models with several prescriptive predictors and possible interactions. These approaches hold value, especially in ML settings, where traditional power analysis approaches would not suffice because of possible complex and non-linear associations between a predictor and outcome [1, 2]. By repeatedly simulating data sets of bigger sample sizes and testing model performance, this method offers a pragmatic and versatile way to compute the minimum required sample size while accounting for the data and model structures [i.e., number of predictors, target AUC, and number of resampling iterations through cross-validation or bootstrapping; 3]. This method concurs with research on the value of simulation-based power analysis in complex multivariable ML settings, providing more individualized estimates of the minimum required sample size relative to traditional approaches [4]. Following this approach, we determined that to detect two-way interactions of Treatment x Predictor in predicting a binary Remission outcome, a sample size of at least 150 was required with 17 predictors in the initial model. The minimum sample size needed with 10 prescriptive predictors in the final model was 100.

**App intervention arms**

**Mindfulness ecological momentary intervention (MEMI).** Multimedia Appendix 3 displays screenshots of MEMI prompts. During each app prompt, MEMI participants were initially asked to engage in *slowed, steady, rhythmic breathing*, “Pay attention to your breathing. Breathe in a slow, steady, and rhythmic manner. Stay focused on the sensations of the air coming into your lungs and then letting it out. Click ‘Continue’”. Then, they were asked to exercise *open monitoring* and *acceptance*, “As you’re breathing, observe your experience as it is. Let go of judgments that do not serve you. Focus your attention in the here-and-now. Click ‘Continue.’” Finally, they were asked to *attend to small moments*: “Attend to the small moments right now (e.g., reading a chapter, having a cool glass of water) as that is where enjoyment, peace, and serenity in life happen. Click ‘Okay’ to continue.”

**Self-monitoring app (SM).** Multimedia Appendix 4 shows screenshots of the app prompts for the SN condition. During each app prompt, SM participants were asked to observe their thoughts, “Notice your thoughts and how distressing they may be. Click ‘Okay’ to continue.”

**Ethical guidelines**

The present study followed stringent ethical principles for psychiatric studies, prioritizing individual well-being and voluntary informed consent. The investigators attended to ethical procedures for studying individuals with mental illnesses, as detailed in the literature [5, 6]. The researchers attained ethics approval from the National University of Singapore (NUS), complying with strict institutional standards. Voluntary informed consent protocols were implemented, offering participants jargon-free, clear, and direct details about the study’s aims, procedures, and possible risks and benefits. Considering the vulnerable population, all research personnel involved in data collection ensured that only willing individuals with the mental capacity to offer informed consent were enrolled in the study. Participants’ confidentiality and privacy were maintained during and after the study’s conclusion using safe, secure, and password-encrypted data storage platforms accessible only to the research team [7]. The investigators also paid particular attention to minimizing possible harms while maximizing potential benefits, which is in line with the ethical principles of beneficence and non-maleficence in psychiatric studies [8, 9].

## Relevance of measures to study objectives

The chosen measures in the current study were especially appropriate for assessing social anxiety disorder (SAD) since measures with strong psychometric properties are necessary for precision mental health. The Social Phobia Diagnostic Questionnaire [SPDQ; 10], Generalized Anxiety Disorder (GAD) Questionnaire-IV [GADQ-IV; 11], and Beck Depression Inventory-Second Edition [BDI-II; 12] offered psychometrically reliable and valid measures of SAD, GAD, and depressive symptoms, respectively. Further, the SPDQ and GADQ-IV had structural equivalence with the Diagnostic and Statistical Manual criteria [13]. The inclusion of measures, such as the Difficulties in Emotion Regulation Scale [DERS; 14], Self-Compassion Scale [SCS; 15], Five-Facet Mindfulness Questionnaire [FFMQ; 16], and Attentional Control Scale [ACS; 17], allows for a comprehensive evaluation of emotion regulation, mindfulness, and attention control skills which are key targets in mindfulness-based interventions (MBIs) for anxiety disorders [18]. The Credibility and Expectancy Questionnaire [CEQ; 19] measures expectancy and credibility, which are established theoretical and empirical digital mental health intervention predictors of treatment outcomes [20]. Taken together, this battery of assessments strongly aligned with the study’s aims to build actionable treatment selection tools while determining prescriptive predictors in persons with SAD given scalable interventions.

## Justification of measure selection over alternatives

The measures above were selected due to their robust psychometric properties and pertinence to SAD and associated concepts. Alternatives such as the Social Phobia Scale [SPS; 21] or the Social Interaction Anxiety Scale [SIAS; 21] were not chosen because of the SPDQ’s better sensitivity to change in randomized controlled trials [18, 22, 23]. The SPDQ also has better alignment with structured psychiatric diagnostic interviews to define SAD remission (absence of SAD diagnosis). The GADQ-IV was selected over alternatives, such as the Penn State Worry Questionnaire [PSWQ; 24], because of its better psychometric properties. For depression, we chose the BDI-II over other options, such as the Patient Health Questionnaire-9 [PHQ-9; 25], because of its more extensive psychometric validation and reliability evidence, including better discriminant validity with anxiety constructs than the PHQ-9 [26-28]. The multidimensional FFMQ had better psychometric reliability and validity than other single-factor measures [29], such as the Mindful Attention Awareness Scale [MAAS; 30]. Similarly, the DERS outperformed other emotion dysregulation measures in many psychometric indices, probably due to its comprehensiveness, flexibility, reliability, and validity [31]. In addition, unlike alternatives such as the emotional flanker and visual probe tasks [32], the ACS showed strong internal consistency and construct validity [32, 33]. The SCS and CEQ were selected due to their non-existent alternatives and psychometric strengths. Moreover, our research team has done several cross-cultural measurement invariance studies on most of these measures that examined transdiagnostic processes in both Singapore and the U.S. These studies highlighted cross-cultural construct compatibility in terms of factor structure, item loadings, intercepts, and even latent factor means and variances of the selected measures [34-36].

## Fidelity to study procedures

Fidelity to the MEMI and SM was retained via standardized video presentations recorded by the principal investigator (NHZ) and delivered by highly trained bachelor’s degree (BA)-level research assistants. The principal investigator received rigorous clinical and research training from the senior author (MGN). All research staff underwent rigorous training to ensure standardization of research procedures. Given the fully self-guided nature of the MEMI and SM without human support, minimal work was needed to monitor fidelity to the MEMI and SM protocols.

## Tracking adherence or engagement

The research team monitored engagement with the MEMI or SM in the PACO app, tracking the number of prompts enrolled participants responded to out of 70 in the 14-day intervention phase. This method aligned with recommended guidelines for assessing engagement in digital mental health interventions [37]. Metrics, such as app usage logs and response rates to momentary encouragements and reminders to practice therapy skills, have been a widespread indicator of user engagement [38].

## Rationale for MEMI and SM

MEMI and SM reflected unique ways to target GAD and SAD symptoms with distinct intervention mechanisms. MEMI comprised empirically supported MBI manual components, focusing on nurturing attentive engagement, open monitoring, and present-mindedness [39]. It is comprised of diaphragmatic breathing retraining, which has evidenced efficacy in alleviating anxiety symptoms and somatic arousal [40]. MEMI participants also learned acceptance, nonjudgment, and the importance of persistent mindfulness practices, which have all been theorized to improve emotion regulation (ER) and self-compassion, and decrease SAD symptoms [23]. Comparatively, SM centered on observing potentially stressful emotions and thought patterns and did not teach participants coping skills via MBI practices and principles. Although SM might raise awareness of internal psychological states, critical MBI elements that would drive therapy improvements (e.g., acceptance, breath retraining, mindfulness) were absent. These core differences in theory and content indicated that MEMI had a greater probability than SM to sustain reductions in anxiety symptoms and associated outcomes [41]. Moreover, SM functioned as a comparator that adjusted for expectancy or placebo effects and regression to the mean [42]. Relatedly, five other digital mental health RCTs have similarly validated the SM as a placebo comparator in targeting GAD [39, 43-46].

## Fully self-guided intervention nature

Research personnel (bachelor’s and master’s degree candidates) trained by the Ph.D.-level principal investigator (NHZ), helped participants to download and install the study app. All study personnel were blinded to participant’s assigned intervention via participants directly viewing of pre-recorded videos that provided training on use of their assigned intervention app and also the rationale for their assigned intervention. Participants then engaged with the assigned MEMI or SM without asynchronously or synchronously contacting any clinically trained therapist or technician during the two-week intervention phase. Please also refer to the section above titled “Fidelity to study procedures.”

References

1. Wilson DT, Hooper R, Brown J, Farrin AJ, Walwyn RE. Efficient and flexible simulation-based sample size determination for clinical trials with multiple design parameters. Stat Methods Med Res. 2021 Mar;30(3):799-815. PMID: 33267735. doi: 10.1177/0962280220975790.

2. Riley RD, Snell KIE, Ensor J, Burke DL, Harrell FE, Jr., Moons KGM, et al. Minimum sample size for developing a multivariable prediction model: Part I - Continuous outcomes. Stat Med. 2019 Mar 30;38(7):1262-75. PMID: 30347470. doi: 10.1002/sim.7993.

3. Riley RD, Snell KI, Ensor J, Burke DL, Harrell FE, Jr., Moons KG, et al. Minimum sample size for developing a multivariable prediction model: PART II - binary and time-to-event outcomes. Stat Med. 2019 Mar 30;38(7):1276-96. PMID: 30357870. doi: 10.1002/sim.7992.

4. Lakens D, Caldwell AR. Simulation-based power analysis for factorial analysis of variance designs. Advances in Methods and Practices in Psychological Science. 2021 2021/01/01;4(1):2515245920951503. doi: 10.1177/2515245920951503.

5. Davies T. Informed consent in psychiatric research. British Journal of Psychiatry. 2001;178(5):397-8. doi: 10.1192/bjp.178.5.397.

6. Roberts LW. The ethical basis of psychiatric research: conceptual issues and empirical findings. Compr Psychiatry. 1998 May-Jun;39(3):99-110. PMID: 9606575. doi: 10.1016/s0010-440x(98)90068-2.

7. Choudhury S, Ghosh A. Ethical considerations of mental health research amidst COVID-19 pandemic: Mitigating the challenges. Indian J Psychol Med. 2020 2020/07/01;42(4):379-81. doi: 10.1177/0253717620929097.

8. Buchanan D, Warwick I. First do no harm: using ‘ethical triage’ to minimise causing harm when undertaking educational research among vulnerable participants. Journal of Further and Higher Education. 2021;45(8):1090-103. doi: 10.1080/0309877x.2021.1890702.

9. Chiumento A, Khan MN, Rahman A, Frith L. Managing ethical challenges to mental health research in post-conflict settings. Dev World Bioeth. 2016 Apr;16(1):15-28. PMID: 25580875. doi: 10.1111/dewb.12076.

10. Newman MG, Kachin KE, Zuellig AR, Constantino MJ, Cashman-McGrath L. The Social Phobia Diagnostic Questionnaire: Preliminary validation of a new self-report diagnostic measure of social phobia. Psychological Medicine. 2003 May;33(4):623-35. PMID: 2003-05628-008. doi: 10.1017/S0033291703007669.

11. Newman MG, Zuellig AR, Kachin KE, Constantino MJ, Przeworski A, Erickson T, et al. Preliminary reliability and validity of the Generalized Anxiety Disorder Questionnaire-IV: A revised self-report diagnostic measure of generalized anxiety disorder. Behavior Therapy. 2002 Spr;33(2):215-33. PMID: 2002-06397-003. doi: 10.1016/S0005-7894(02)80026-0.

12. Beck A, Steer R, Brown G. Beck Depression Inventory. Second ed. San Antonio, TX: Psychological Corporation; 1996.

13. American Psychiatric Association. Diagnostic and Statistical Manual of Mental Disorders (DSM-5). DSM-5-TR ed: American Psychiatric Association Publishing; 2022 2022/03/18/. ISBN: 978-0-89042-575-6.

14. Gratz KL, Roemer L. Multidimensional assessment of emotion regulation and dysregulation: Development, factor structure, and initial validation of the difficulties in emotion regulation scale. Journal of Psychopathology and Behavioral Assessment. 2004;26(1):41-54. doi: 10.1023/B:JOBA.0000007455.08539.94.

15. Neff KD. The development and validation of a scale to measure self-compassion. Self and Identity. 2003 2003/07/01;2(3):223-50. doi: 10.1080/15298860309027.

16. Baer RA, Smith GT, Lykins E, Button D, Krietemeyer J, Sauer S, et al. Construct validity of the five facet mindfulness questionnaire in meditating and nonmeditating samples. Assessment. 2008 Sep;15(3):329-42. PMID: 18310597. doi: 10.1177/1073191107313003.

17. Derryberry D, Reed MA. Anxiety-related attentional biases and their regulation by attentional control. Journal of Abnormal Psychology. 2002;111(2):225-36. doi: 10.1037/0021-843X.111.2.225.

18. Zainal NH, Tan HH, Hong RYS, Newman MG. Testing the efficacy of a brief, self-guided mindfulness ecological momentary intervention on emotion regulation and self-compassion in social anxiety disorder: Randomized controlled trial. JMIR Ment Health. 2024 2024/4/19;11:e53712. doi: 10.2196/53712.

19. Devilly GJ, Borkovec TD. Psychometric properties of the credibility/expectancy questionnaire. Journal of Behavior Therapy and Experimental Psychiatry. 2000;31(2):73-86. doi: 10.1016/S0005-7916(00)00012-4.

20. Ponten M, Jonsjo M, Vadenmark V, Moberg E, Grannas D, Andersson G, et al. Association between expectations and clinical outcomes in online v. face-to-face therapy - an individual participant data meta-analysis. Psychol Med. 2024 Apr;54(6):1207-14. PMID: 37905404. doi: 10.1017/S0033291723003033.

21. Mattick RP, Clarke JC. Development and validation of measures of social phobia scrutiny fear and social interaction anxiety. Behaviour Research and Therapy. 1998;36(4):455-70. doi: 10.1016/S0005-7967(97)10031-6.

22. Zainal NH, Chan WW, Saxena AP, Taylor CB, Newman MG. Pilot randomized trial of self-guided virtual reality exposure therapy for social anxiety disorder. Behaviour Research and Therapy. 2021;147:103984. doi: 10.1016/j.brat.2021.103984.

23. Zainal NH, Tan HH, Hong RY, Newman MG. Is a brief mindfulness ecological momentary intervention more efficacious than a self-monitoring app for social anxiety disorder? A randomized controlled trial. J Anxiety Disord. 2024 Jun;104:102858. PMID: 38657408. doi: 10.1016/j.janxdis.2024.102858.

24. Meyer TJ, Miller ML, Metzger RL, Borkovec TD. Development and validation of the Penn State Worry Questionnaire. Behaviour Research and Therapy. 1990 Dec;28(6):487-95. doi: 10.1016/0005-7967(90)90135-6.

25. Kroenke K, Spitzer RL. The PHQ-9: A new depression diagnostic and severity measure. Psychiatric Annals. 2002;32(9):509-15. doi: 10.3928/0048-5713-20020901-06.

26. García-Batista ZE, Guerra-Peña K, Cano-Vindel A, Herrera-Martínez SX, Medrano LA. Validity and reliability of the Beck Depression Inventory (BDI-II) in general and hospital population of Dominican Republic. PLoS ONE. 2018;13(6):e0199750. PMID: 29958268. doi: 10.1371/journal.pone.0199750.

27. Huang SL, Hsieh CL, Wu RM, Lu WS. Test-retest reliability and minimal detectable change of the Beck Depression Inventory and the Taiwan Geriatric Depression Scale in patients with Parkinson's disease. PLoS ONE. 2017;12(9):e0184823. PMID: 28945776. doi: 10.1371/journal.pone.0184823.

28. Wang Y-P, Gorenstein C. Psychometric properties of the Beck Depression Inventory-II: A comprehensive review. Revista Brasileira de Psiquiatria. 2013 Oct-Dec;35(4):416-31. PMID: 24402217. doi: 10.1590/1516-4446-2012-1048.

29. MacKillop J, Anderson EJ. Further psychometric validation of the Mindful Attention Awareness Scale (MAAS). Journal of Psychopathology and Behavioral Assessment. 2007 2007/12/01;29(4):289-93. doi: 10.1007/s10862-007-9045-1.

30. Brown KW, Ryan RM. The benefits of being present: mindfulness and its role in psychological well-being. J Pers Soc Psychol. 2003 Apr;84(4):822-48. PMID: 12703651. doi: 10.1037/0022-3514.84.4.822.

31. Sorman K, Garke MA, Isacsson NH, Jangard S, Bjureberg J, Hellner C, et al. Measures of emotion regulation: Convergence and psychometric properties of the Difficulties in Emotion Regulation Scale and Emotion Regulation Questionnaire. J Clin Psychol. 2022 Feb;78(2):201-17. PMID: 34217149. doi: 10.1002/jclp.23206.

32. Van Bockstaele B, Lamens L, Salemink E, Wiers RW, Bogels SM, Nikolaou K. Reliability and validity of measures of attentional bias towards threat in unselected student samples: seek, but will you find? Cogn Emot. 2020 Mar;34(2):217-28. PMID: 31044648. doi: 10.1080/02699931.2019.1609423.

33. Ishikawa H, Koshikawa F. Self-reported attention control skills moderate the effect of self-focused attention on depression. Sage Open. 2021 2021/04/01;11(2):21582440211027965. doi: 10.1177/21582440211027965.

34. Van Doren N, Zainal NH, Newman MG. Cross-cultural and gender invariance of emotion regulation in the United States and India. Journal of Affective Disorders. 2021 Dec 1;295:1360–70. PMID: 34706449. doi: 10.1016/j.jad.2021.04.089.

35. Van Doren N, Zainal NH, Newman MG, Hong RY. Cross-cultural and gender invariance of six common symptom and cognitive vulnerability measures in the United States and Singapore. Cognitive Therapy and Research. in press. doi: 10.1007.s10608-024-10519-4.

36. Zainal NH, Newman MG, Hong RY. Cross-cultural and gender invariance of transdiagnostic processes in the United States and Singapore. Assessment. 2021;28:485-502. PMID: 31538795. doi: 10.1177/1073191119869832.

37. Perski O, Blandford A, West R, Michie S. Conceptualising engagement with digital behaviour change interventions: a systematic review using principles from critical interpretive synthesis. Transl Behav Med. 2017 Jun;7(2):254-67. PMID: 27966189. doi: 10.1007/s13142-016-0453-1.

38. Torous J, Nicholas J, Larsen ME, Firth J, Christensen H. Clinical review of user engagement with mental health smartphone apps: evidence, theory and improvements. Evid Based Ment Health. 2018 Aug;21(3):116-9. PMID: 29871870. doi: 10.1136/eb-2018-102891.

39. Zainal NH, Newman MG. A randomized controlled trial of a 14-day mindfulness ecological momentary intervention (MEMI) for generalized anxiety disorder. European Psychiatry. 2023 Jan 16;66(1):e12. PMID: 36645098. doi: 10.1192/j.eurpsy.2023.2.

40. Hopper SI, Murray SL, Ferrara LR, Singleton JK. Effectiveness of diaphragmatic breathing for reducing physiological and psychological stress in adults: a quantitative systematic review. JBI Database System Rev Implement Rep. 2019 Sep;17(9):1855-76. PMID: 31436595. doi: 10.11124/JBISRIR-2017-003848.

41. Wang K, Varma DS, Prosperi M. A systematic review of the effectiveness of mobile apps for monitoring and management of mental health symptoms or disorders. J Psychiatr Res. 2018 Dec;107:73-8. PMID: 30347316. doi: 10.1016/j.jpsychires.2018.10.006.

42. Faurholt-Jepsen M, Munkholm K, Frost M, Bardram JE, Kessing LV. Electronic self-monitoring of mood using IT platforms in adult patients with bipolar disorder: A systematic review of the validity and evidence. BMC Psychiatry. 2016 Jan 15;16:7. PMID: 26769120. doi: 10.1186/s12888-016-0713-0.

43. LaFreniere LS, Newman MG. A brief ecological momentary intervention for Generalized Anxiety Disorder: A randomized controlled trial of the worry outcome journal. Depression and Anxiety. 2016;33(9):829-39. doi: 10.1002/da.22507.

44. LaFreniere LS, Newman MG. The impact of uncontrollability beliefs and thought-related distress on ecological momentary interventions for generalized anxiety disorder: A moderated mediation model. Journal of anxiety disorders. 2019 Aug;66:102113. PMID: 31362145. doi: 10.1016/j.janxdis.2019.102113.

45. LaFreniere LS, Newman MG. Exposing worry’s deceit: Percentage of untrue worries in generalized anxiety disorder treatment. Behavior Therapy. 2020 May;51(3):413-23. PMID: 32402257. doi: 10.1016/j.beth.2019.07.003.

46. LaFreniere LS, Newman MG. Upregulating positive emotion in generalized anxiety disorder: A randomized controlled trial of the SkillJoy ecological momentary intervention. J Consult Clin Psychol. 2023 Jun;91(6):381-7. PMID: 36716146. doi: 10.1037/ccp0000794.

47. Sajjadian M, Uher R, Ho K, Hassel S, Milev R, Frey BN, et al. Prediction of depression treatment outcome from multimodal data: a CAN-BIND-1 report. Psychol Med. 2023 Sep;53(12):5374-84. PMID: 36004538. doi: 10.1017/S0033291722002124.

48. Liu S, Schlesinger JJ, McCoy AB, Reese TJ, Steitz B, Russo E, et al. New onset delirium prediction using machine learning and long short-term memory (LSTM) in electronic health record. J Am Med Inform Assoc. 2022 Dec 13;30(1):120-31. PMID: 36303456. doi: 10.1093/jamia/ocac210.

49. Islam R, Layek MA. StackEnsembleMind: Enhancing well-being through accurate identification of human mental states using stack-based ensemble machine learning. Informatics in Medicine Unlocked. 2023;43. doi: 10.1016/j.imu.2023.101405.

50. Varma S, Simon R. Bias in error estimation when using cross-validation for model selection. BMC Bioinformatics. 2006 Feb 23;7:Article 91. PMID: 16504092. doi: 10.1186/1471-2105-7-91.

51. Cawley GC, Talbot NLC. On over-fitting in model selection and subsequent selection bias in performance evaluation. Journal of Machine Learning Research. 2010 Jul;11(70):2079–107. PMID: WOS:000282523000006.

52. Cortes C, Vapnik V. Support-vector networks. Machine Learning. 1995 1995/09/01;20(3):273-97. doi: 10.1007/BF00994018.

53. Rothacher Y, Strobl C. Identifying informative predictor variables with random forests. Journal of Educational and Behavioral Statistics. 2023;49(4):595-629. doi: 10.3102/10769986231193327.

54. Breiman L. Random forests. Machine Learning. 2001;45(1):5-32. doi: 10.1023/A:1010933404324.

55. Guenther N, Schonlau M. Support vector machines. The Stata Journal. 2016 2016/12/01;16(4):917-37. doi: 10.1177/1536867X1601600407.

56. Hastie T, Tibshirani R, Friedman J, editors. The elements of statistical learning. New York, NY: Springer Science & Business Media; 2009.

57. Hernán MA, Robins JM. Causal inference: What if. Boca Raton: Chapman & Hall/CRC; 2023.

58. Imbens GW, Rubin DB. Causal inference for statistics, social, and biomedical sciences: An introduction. New York, NY, US: Cambridge University Press; 2015. xix, 625-xix, p. ISBN: 978-0-521-88588-1 (Hardcover).

59. Austin PC, Stuart EA. Moving towards best practice when using inverse probability of treatment weighting (IPTW) using the propensity score to estimate causal treatment effects in observational studies. Stat Med. 2015 Dec 10;34(28):3661-79. PMID: 26238958. doi: 10.1002/sim.6607.

60. VanderWeele TJ. On the distinction between interaction and effect modification. Epidemiology. 2009 Nov;20(6):863-71. PMID: 19806059. doi: 10.1097/EDE.0b013e3181ba333c.
