# Supplementary figures and images for: Prescriptive Predictors of Mindfulness Ecological Momentary Intervention for Social Anxiety Disorder: Machine Learning Analysis of Randomized Controlled Trial Data
Source: JMIR Ment Health. 2025 May 13;12:e67210. doi: 10.2196/67210 (PMC12117280; doi:10.2196/67210)

# Multimedia Appendix 4

## Screenshots for self-monitoring app (SM) arm


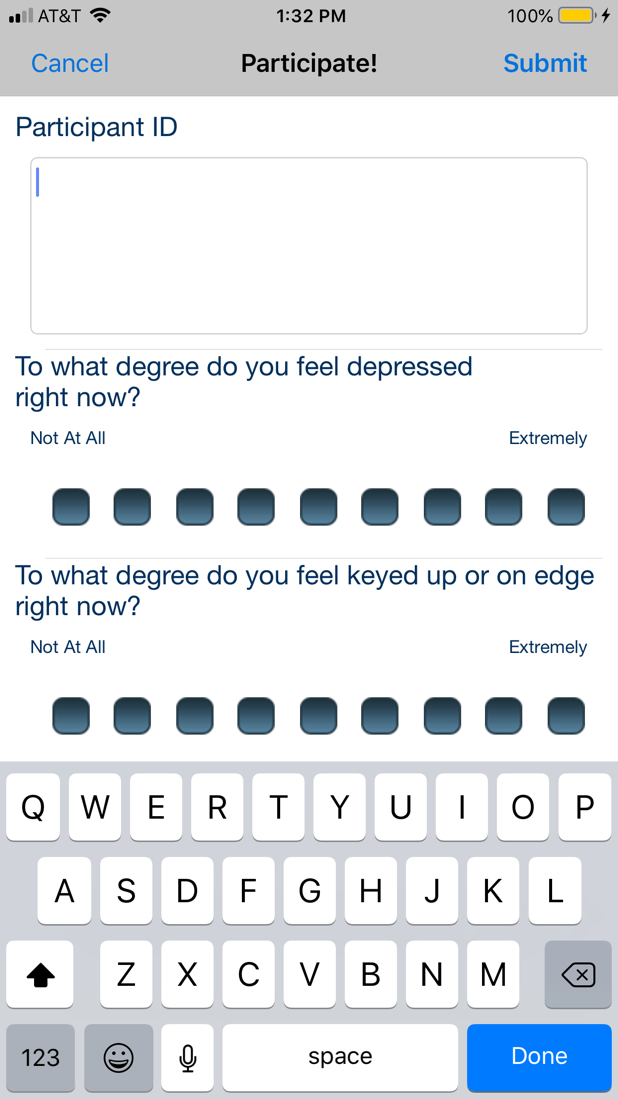

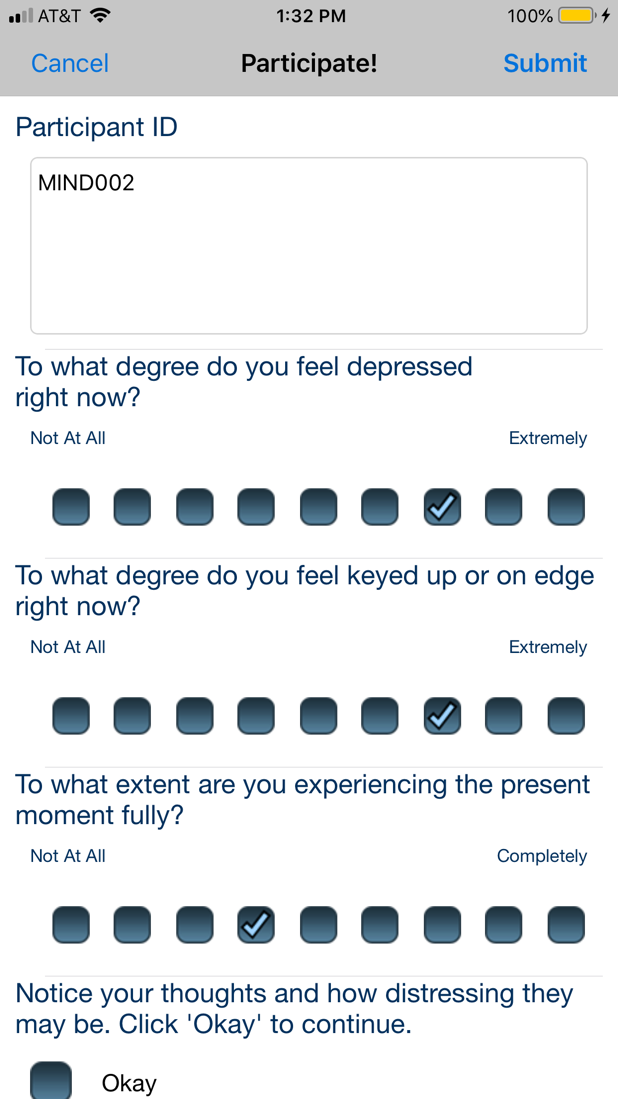


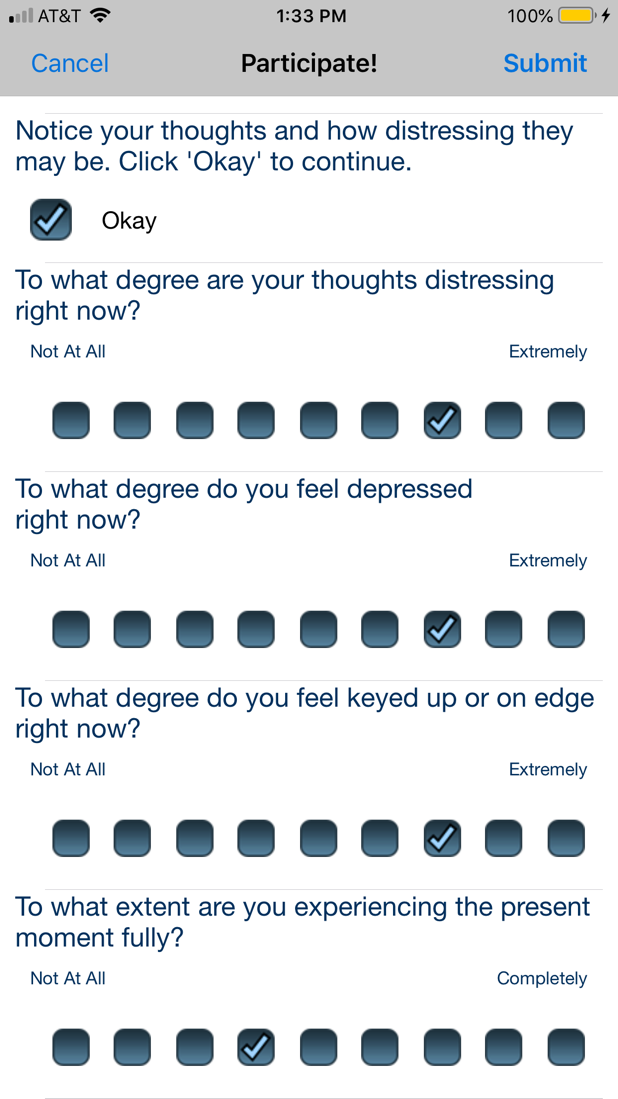

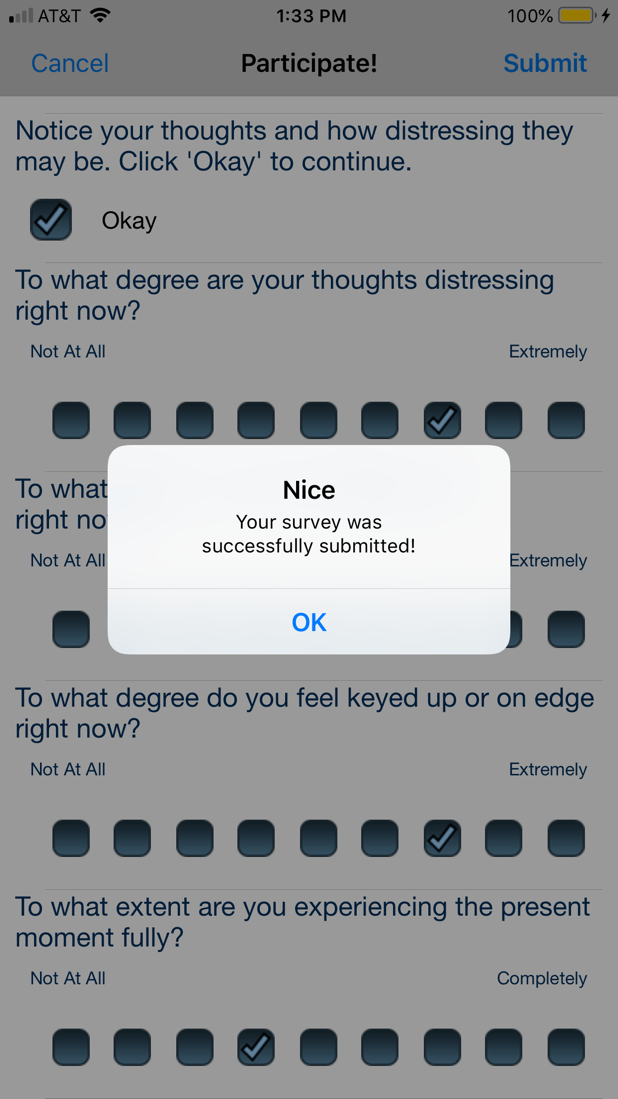

Supplement: Multimedia Appendix 4 [file mental_v12i1e67210_app4.docx]
