# Supplementary material for: Prescriptive Predictors of Mindfulness Ecological Momentary Intervention for Social Anxiety Disorder: Machine Learning Analysis of Randomized Controlled Trial Data
Source: JMIR Ment Health. 2025 May 13;12:e67210. doi: 10.2196/67210 (PMC12117280; doi:10.2196/67210)
Supplement: Multimedia Appendix 5 [file mental_v12i1e67210_app5.docx]

# Multimedia Appendix 5

## Rationale for machine learning (ML) approach

Random forest (RF) and support vector machine (SVM) were selected because they often showed model performance that superseded logistic regression in precision mental health because of their capacity to model complex, non-linear associations and higher-order interactions in high-dimensional data sets. Myriad studies suggested the benefits of these ML methods in predicting clinical outcomes. For example, a study showed that RF ensemble models attained the most optimal model performance in predicting depression outcomes, with a mean balanced accuracy of .62 relative to .54 in non-ensemble models [47]. Similarly, SVM has been shown to have better model performance than logistic regression in predicting incident cases of delirium with an area under the receiver operating characteristic curve (AUC) value of .93 relative to lower model performance values with other algorithms [48]. A systematic review also evidenced that RF and SVM typically presented with better model performance metrics than other ML models for psychiatric prediction tasks [49]. Together, these ML approaches provided enhanced predictive precision, rendering them invaluable in building actionable treatment selection tools.

## Details of multivariable ML modeling approach

The analysis used default tuning parameters, as we used nested 10-fold cross-validation (10F-CV) with 10 repetitions to adjust for overfitting and generate stronger model performance metrics [50]. This method was well-suited for maximizing data usage and minimizing biased estimates for small samples [51]. Overfitting (lack-of-generalizability problems) was addressed using RF and SVM algorithms, which effectively manage high-dimensional, sparse data sets with large ratios between the number of predictors and sample size [52]. RF has been shown to generate strong performance with default hyperparameters, given its lower sensitivity to parameter tuning relative to other ML algorithms [53]. RF thus provided decreased vulnerability to overfitting and reduced effects of outliers relative to conventional decision trees [54]. Similarly, SVM could attain acceptable ML performance without excessive hyperparameter tuning, particularly given how the present study used the radial basis function kernelling approach [55]. The nested CV structure, with inner folds for model selection with default tuning parameters and training and outer folds for testing, ensured that model testing metrics were derived from the unseen test folds. This approach optimized the probability of creating generalizable (reliable) estimates without tuning [56].

## Assumptions of the causal inference approach to study heterogeneous treatment effects

The counterfactual causal inference approach relies on several critical assumptions, including conditional exchangeability (no unmeasured confounding), consistency, and positivity [57]. First, the original randomized controlled trial (RCT) design helps support the conditional exchangeability assumption by balancing measured and unmeasured confounders between treatment arms [58]. Moreover, none of the 17 variables used as potential prescriptive predictors were statistically different across the two arms (*p* = .10 to .96), indicating conditional exchangeability for measured confounders.

The positivity assumption required that each participant had a non-zero probability of receiving either treatment [59]. This assumption is likely satisfied by our randomized study design and the observation of sufficient overlap between treatment arms when checking for extreme propensity scores (cf. Figures S1 and S3 below).

**Figure S1**

*Histogram of propensity scores*


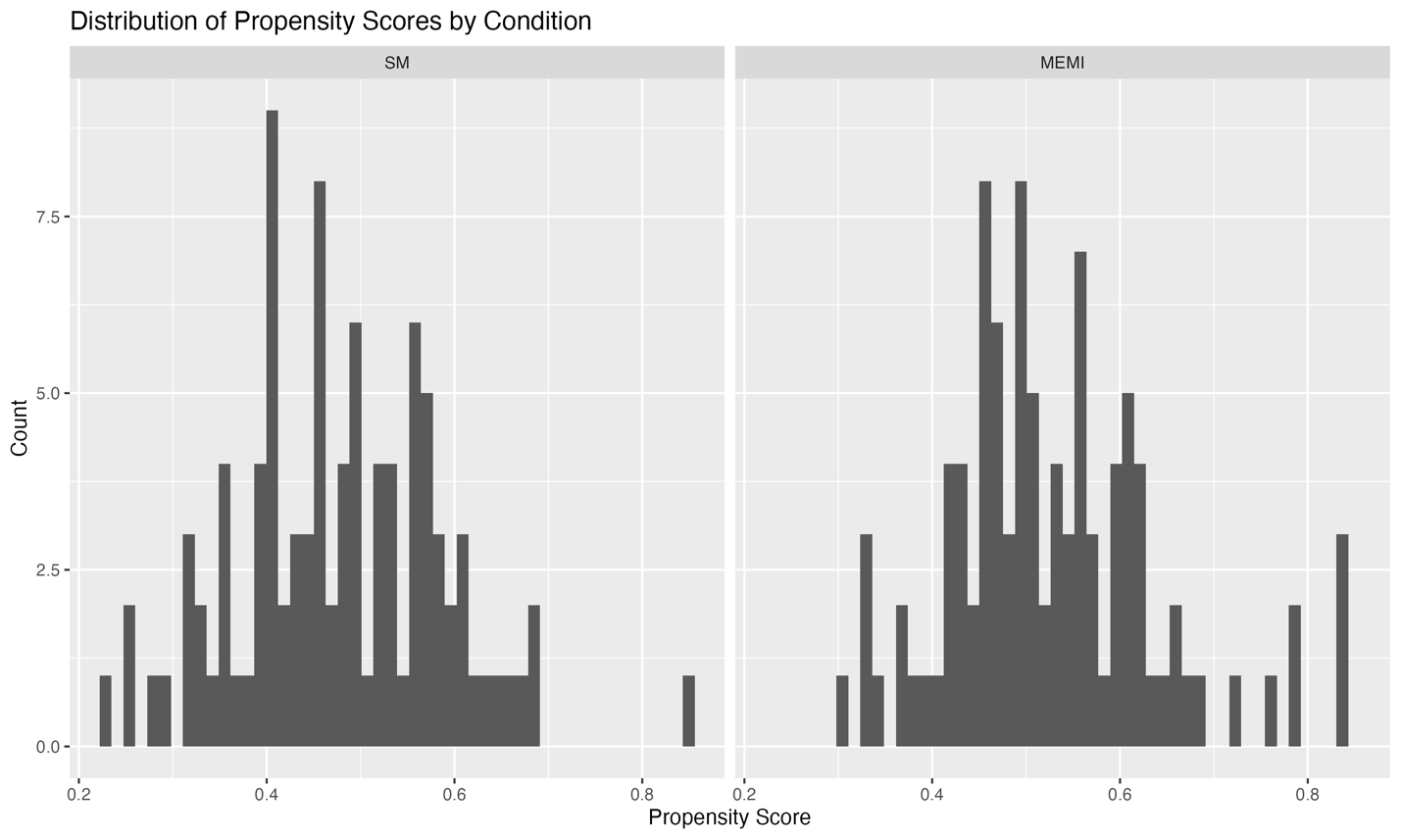


*Note.* SM, self-monitoring app; MEMI, mindfulness ecological momentary intervention.

**Figure S2**

*Density plot of propensity scores (untrimmed)*


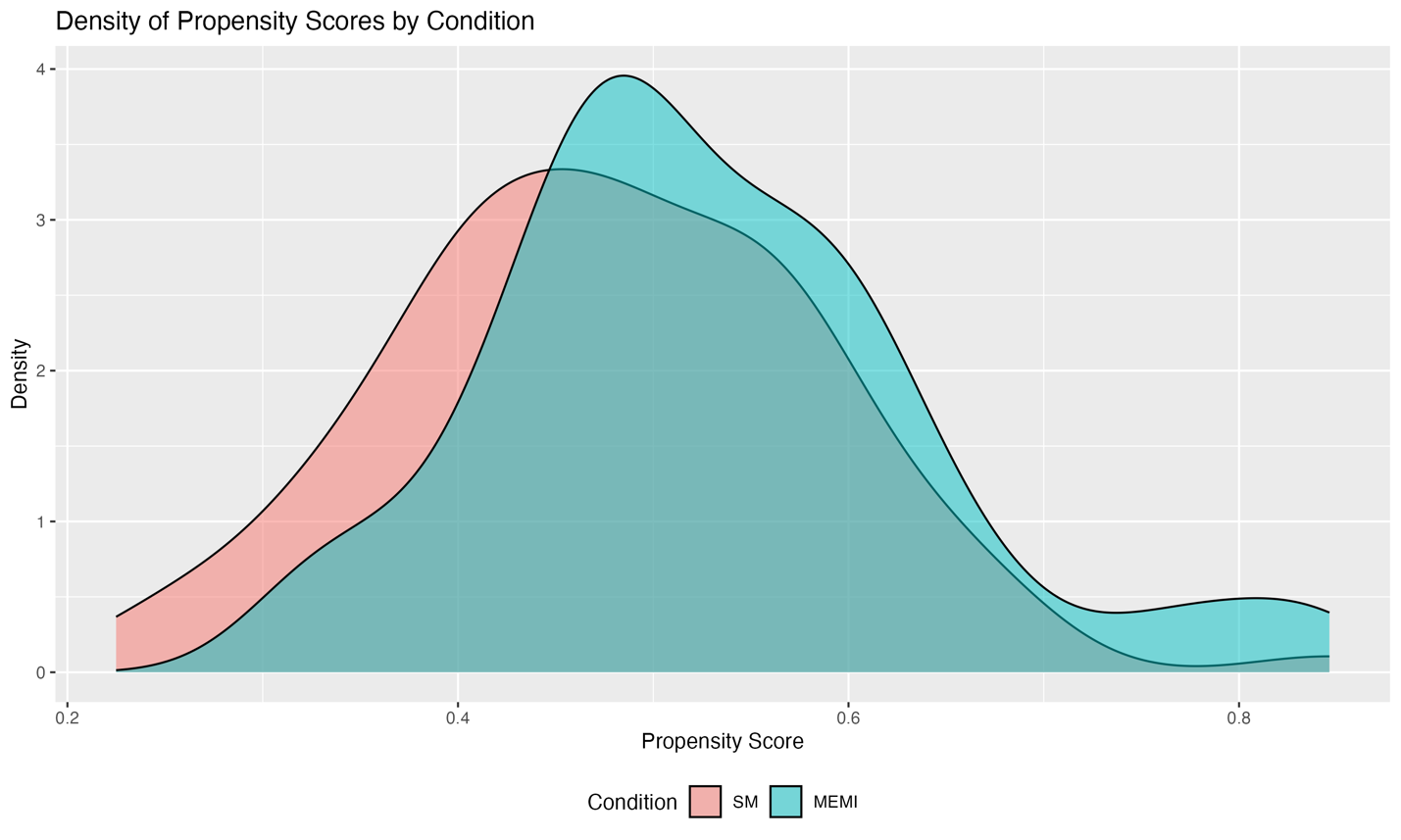


*Note.* SM, self-monitoring app; MEMI, mindfulness ecological momentary intervention.

**Figure S3**

*Density plot of propensity scores (trimmed)*


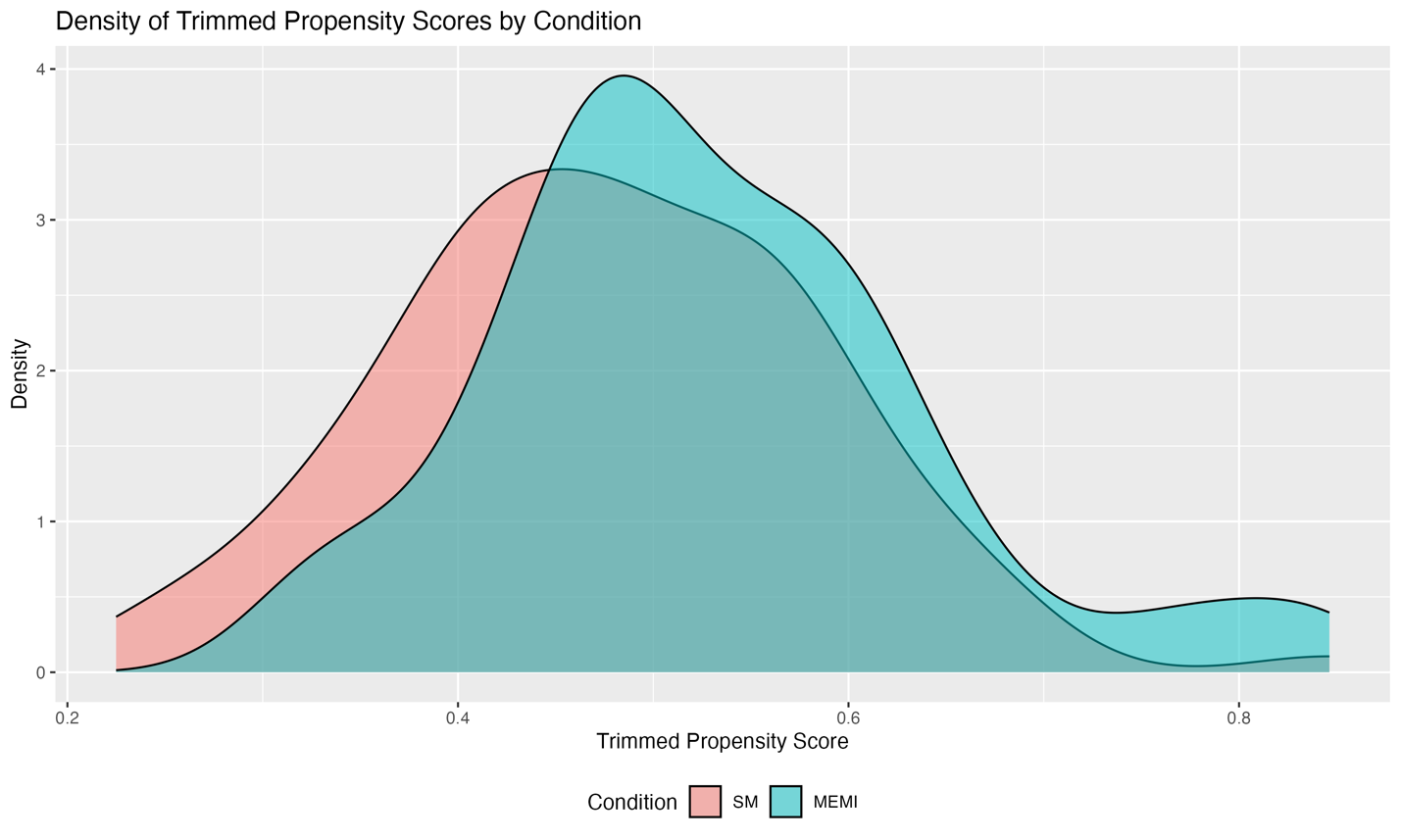


*Note.* SM, self-monitoring app; MEMI, mindfulness ecological momentary intervention.

The consistency assumption posits that the observed outcome under the received treatment is equal to the potential outcome under that treatment [60]. This is generally assumed to hold for well-defined interventions and the fact that there were no or minimal concerns about treatment variations or contamination across the MEMI and SM.

**Calibration Plots**

This analysis performed a calibration assessment for two predictive models: a pre-post model and a pre-1MFU (baseline to 1-month follow-up) model. The calibration analysis function evaluated how well the predicted probabilities from each model align with the observed outcomes. It generated calibration plots (see Figures S4 and S5 below), which visually represented this alignment. It calculated two key metrics: the Brier score (a measure of prediction accuracy, with lower scores indicating better calibration) and the area under the curve (AUC, a measure of discriminative ability). The results showed that both models have moderate discriminative ability (AUC ranging from .739 to .744) but suboptimal calibration (high Brier scores of 1.925-1.971). This analysis provided insights into the models’ performance in predicting treatment outcomes, which is crucial for clinical psychologists considering the implementation of these predictive tools in practice.

**Figure S4**

*Calibration plot for the pre-post outcome model*


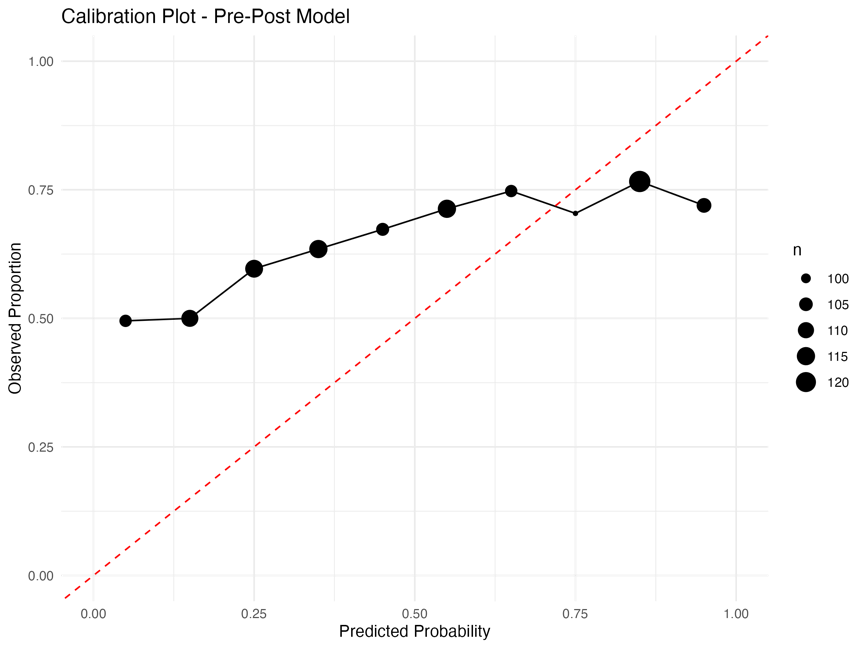


**Figure S5**

*Calibration plot for the pre-1MFU outcome model*


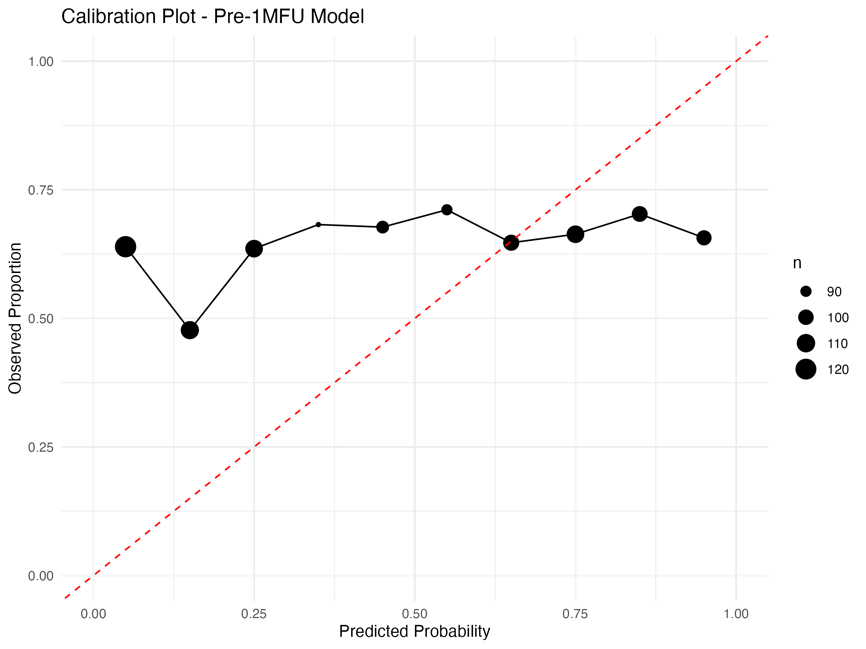


*Note.* 1MFU, one-month follow-up.

References

1. Wilson DT, Hooper R, Brown J, Farrin AJ, Walwyn RE. Efficient and flexible simulation-based sample size determination for clinical trials with multiple design parameters. Stat Methods Med Res. 2021 Mar;30(3):799-815. PMID: 33267735. doi: 10.1177/0962280220975790.

2. Riley RD, Snell KIE, Ensor J, Burke DL, Harrell FE, Jr., Moons KGM, et al. Minimum sample size for developing a multivariable prediction model: Part I - Continuous outcomes. Stat Med. 2019 Mar 30;38(7):1262-75. PMID: 30347470. doi: 10.1002/sim.7993.

3. Riley RD, Snell KI, Ensor J, Burke DL, Harrell FE, Jr., Moons KG, et al. Minimum sample size for developing a multivariable prediction model: PART II - binary and time-to-event outcomes. Stat Med. 2019 Mar 30;38(7):1276-96. PMID: 30357870. doi: 10.1002/sim.7992.

4. Lakens D, Caldwell AR. Simulation-based power analysis for factorial analysis of variance designs. Advances in Methods and Practices in Psychological Science. 2021 2021/01/01;4(1):2515245920951503. doi: 10.1177/2515245920951503.

5. Davies T. Informed consent in psychiatric research. British Journal of Psychiatry. 2001;178(5):397-8. doi: 10.1192/bjp.178.5.397.

6. Roberts LW. The ethical basis of psychiatric research: conceptual issues and empirical findings. Compr Psychiatry. 1998 May-Jun;39(3):99-110. PMID: 9606575. doi: 10.1016/s0010-440x(98)90068-2.

7. Choudhury S, Ghosh A. Ethical considerations of mental health research amidst COVID-19 pandemic: Mitigating the challenges. Indian J Psychol Med. 2020 2020/07/01;42(4):379-81. doi: 10.1177/0253717620929097.

8. Buchanan D, Warwick I. First do no harm: using ‘ethical triage’ to minimise causing harm when undertaking educational research among vulnerable participants. Journal of Further and Higher Education. 2021;45(8):1090-103. doi: 10.1080/0309877x.2021.1890702.

9. Chiumento A, Khan MN, Rahman A, Frith L. Managing ethical challenges to mental health research in post-conflict settings. Dev World Bioeth. 2016 Apr;16(1):15-28. PMID: 25580875. doi: 10.1111/dewb.12076.

10. Newman MG, Kachin KE, Zuellig AR, Constantino MJ, Cashman-McGrath L. The Social Phobia Diagnostic Questionnaire: Preliminary validation of a new self-report diagnostic measure of social phobia. Psychological Medicine. 2003 May;33(4):623-35. PMID: 2003-05628-008. doi: 10.1017/S0033291703007669.

11. Newman MG, Zuellig AR, Kachin KE, Constantino MJ, Przeworski A, Erickson T, et al. Preliminary reliability and validity of the Generalized Anxiety Disorder Questionnaire-IV: A revised self-report diagnostic measure of generalized anxiety disorder. Behavior Therapy. 2002 Spr;33(2):215-33. PMID: 2002-06397-003. doi: 10.1016/S0005-7894(02)80026-0.

12. Beck A, Steer R, Brown G. Beck Depression Inventory. Second ed. San Antonio, TX: Psychological Corporation; 1996.

13. American Psychiatric Association. Diagnostic and Statistical Manual of Mental Disorders (DSM-5). DSM-5-TR ed: American Psychiatric Association Publishing; 2022 2022/03/18/. ISBN: 978-0-89042-575-6.

14. Gratz KL, Roemer L. Multidimensional assessment of emotion regulation and dysregulation: Development, factor structure, and initial validation of the difficulties in emotion regulation scale. Journal of Psychopathology and Behavioral Assessment. 2004;26(1):41-54. doi: 10.1023/B:JOBA.0000007455.08539.94.

15. Neff KD. The development and validation of a scale to measure self-compassion. Self and Identity. 2003 2003/07/01;2(3):223-50. doi: 10.1080/15298860309027.

16. Baer RA, Smith GT, Lykins E, Button D, Krietemeyer J, Sauer S, et al. Construct validity of the five facet mindfulness questionnaire in meditating and nonmeditating samples. Assessment. 2008 Sep;15(3):329-42. PMID: 18310597. doi: 10.1177/1073191107313003.

17. Derryberry D, Reed MA. Anxiety-related attentional biases and their regulation by attentional control. Journal of Abnormal Psychology. 2002;111(2):225-36. doi: 10.1037/0021-843X.111.2.225.

18. Zainal NH, Tan HH, Hong RYS, Newman MG. Testing the efficacy of a brief, self-guided mindfulness ecological momentary intervention on emotion regulation and self-compassion in social anxiety disorder: Randomized controlled trial. JMIR Ment Health. 2024 2024/4/19;11:e53712. doi: 10.2196/53712.

19. Devilly GJ, Borkovec TD. Psychometric properties of the credibility/expectancy questionnaire. Journal of Behavior Therapy and Experimental Psychiatry. 2000;31(2):73-86. doi: 10.1016/S0005-7916(00)00012-4.

20. Ponten M, Jonsjo M, Vadenmark V, Moberg E, Grannas D, Andersson G, et al. Association between expectations and clinical outcomes in online v. face-to-face therapy - an individual participant data meta-analysis. Psychol Med. 2024 Apr;54(6):1207-14. PMID: 37905404. doi: 10.1017/S0033291723003033.

21. Mattick RP, Clarke JC. Development and validation of measures of social phobia scrutiny fear and social interaction anxiety. Behaviour Research and Therapy. 1998;36(4):455-70. doi: 10.1016/S0005-7967(97)10031-6.

22. Zainal NH, Chan WW, Saxena AP, Taylor CB, Newman MG. Pilot randomized trial of self-guided virtual reality exposure therapy for social anxiety disorder. Behaviour Research and Therapy. 2021;147:103984. doi: 10.1016/j.brat.2021.103984.

23. Zainal NH, Tan HH, Hong RY, Newman MG. Is a brief mindfulness ecological momentary intervention more efficacious than a self-monitoring app for social anxiety disorder? A randomized controlled trial. J Anxiety Disord. 2024 Jun;104:102858. PMID: 38657408. doi: 10.1016/j.janxdis.2024.102858.

24. Meyer TJ, Miller ML, Metzger RL, Borkovec TD. Development and validation of the Penn State Worry Questionnaire. Behaviour Research and Therapy. 1990 Dec;28(6):487-95. doi: 10.1016/0005-7967(90)90135-6.

25. Kroenke K, Spitzer RL. The PHQ-9: A new depression diagnostic and severity measure. Psychiatric Annals. 2002;32(9):509-15. doi: 10.3928/0048-5713-20020901-06.

26. García-Batista ZE, Guerra-Peña K, Cano-Vindel A, Herrera-Martínez SX, Medrano LA. Validity and reliability of the Beck Depression Inventory (BDI-II) in general and hospital population of Dominican Republic. PLoS ONE. 2018;13(6):e0199750. PMID: 29958268. doi: 10.1371/journal.pone.0199750.

27. Huang SL, Hsieh CL, Wu RM, Lu WS. Test-retest reliability and minimal detectable change of the Beck Depression Inventory and the Taiwan Geriatric Depression Scale in patients with Parkinson's disease. PLoS ONE. 2017;12(9):e0184823. PMID: 28945776. doi: 10.1371/journal.pone.0184823.

28. Wang Y-P, Gorenstein C. Psychometric properties of the Beck Depression Inventory-II: A comprehensive review. Revista Brasileira de Psiquiatria. 2013 Oct-Dec;35(4):416-31. PMID: 24402217. doi: 10.1590/1516-4446-2012-1048.

29. MacKillop J, Anderson EJ. Further psychometric validation of the Mindful Attention Awareness Scale (MAAS). Journal of Psychopathology and Behavioral Assessment. 2007 2007/12/01;29(4):289-93. doi: 10.1007/s10862-007-9045-1.

30. Brown KW, Ryan RM. The benefits of being present: mindfulness and its role in psychological well-being. J Pers Soc Psychol. 2003 Apr;84(4):822-48. PMID: 12703651. doi: 10.1037/0022-3514.84.4.822.

31. Sorman K, Garke MA, Isacsson NH, Jangard S, Bjureberg J, Hellner C, et al. Measures of emotion regulation: Convergence and psychometric properties of the Difficulties in Emotion Regulation Scale and Emotion Regulation Questionnaire. J Clin Psychol. 2022 Feb;78(2):201-17. PMID: 34217149. doi: 10.1002/jclp.23206.

32. Van Bockstaele B, Lamens L, Salemink E, Wiers RW, Bogels SM, Nikolaou K. Reliability and validity of measures of attentional bias towards threat in unselected student samples: seek, but will you find? Cogn Emot. 2020 Mar;34(2):217-28. PMID: 31044648. doi: 10.1080/02699931.2019.1609423.

33. Ishikawa H, Koshikawa F. Self-reported attention control skills moderate the effect of self-focused attention on depression. Sage Open. 2021 2021/04/01;11(2):21582440211027965. doi: 10.1177/21582440211027965.

34. Van Doren N, Zainal NH, Newman MG. Cross-cultural and gender invariance of emotion regulation in the United States and India. Journal of Affective Disorders. 2021 Dec 1;295:1360–70. PMID: 34706449. doi: 10.1016/j.jad.2021.04.089.

35. Van Doren N, Zainal NH, Newman MG, Hong RY. Cross-cultural and gender invariance of six common symptom and cognitive vulnerability measures in the United States and Singapore. Cognitive Therapy and Research. in press. doi: 10.1007.s10608-024-10519-4.

36. Zainal NH, Newman MG, Hong RY. Cross-cultural and gender invariance of transdiagnostic processes in the United States and Singapore. Assessment. 2021;28:485-502. PMID: 31538795. doi: 10.1177/1073191119869832.

37. Perski O, Blandford A, West R, Michie S. Conceptualising engagement with digital behaviour change interventions: a systematic review using principles from critical interpretive synthesis. Transl Behav Med. 2017 Jun;7(2):254-67. PMID: 27966189. doi: 10.1007/s13142-016-0453-1.

38. Torous J, Nicholas J, Larsen ME, Firth J, Christensen H. Clinical review of user engagement with mental health smartphone apps: evidence, theory and improvements. Evid Based Ment Health. 2018 Aug;21(3):116-9. PMID: 29871870. doi: 10.1136/eb-2018-102891.

39. Zainal NH, Newman MG. A randomized controlled trial of a 14-day mindfulness ecological momentary intervention (MEMI) for generalized anxiety disorder. European Psychiatry. 2023 Jan 16;66(1):e12. PMID: 36645098. doi: 10.1192/j.eurpsy.2023.2.

40. Hopper SI, Murray SL, Ferrara LR, Singleton JK. Effectiveness of diaphragmatic breathing for reducing physiological and psychological stress in adults: a quantitative systematic review. JBI Database System Rev Implement Rep. 2019 Sep;17(9):1855-76. PMID: 31436595. doi: 10.11124/JBISRIR-2017-003848.

41. Wang K, Varma DS, Prosperi M. A systematic review of the effectiveness of mobile apps for monitoring and management of mental health symptoms or disorders. J Psychiatr Res. 2018 Dec;107:73-8. PMID: 30347316. doi: 10.1016/j.jpsychires.2018.10.006.

42. Faurholt-Jepsen M, Munkholm K, Frost M, Bardram JE, Kessing LV. Electronic self-monitoring of mood using IT platforms in adult patients with bipolar disorder: A systematic review of the validity and evidence. BMC Psychiatry. 2016 Jan 15;16:7. PMID: 26769120. doi: 10.1186/s12888-016-0713-0.

43. LaFreniere LS, Newman MG. A brief ecological momentary intervention for Generalized Anxiety Disorder: A randomized controlled trial of the worry outcome journal. Depression and Anxiety. 2016;33(9):829-39. doi: 10.1002/da.22507.

44. LaFreniere LS, Newman MG. The impact of uncontrollability beliefs and thought-related distress on ecological momentary interventions for generalized anxiety disorder: A moderated mediation model. Journal of anxiety disorders. 2019 Aug;66:102113. PMID: 31362145. doi: 10.1016/j.janxdis.2019.102113.

45. LaFreniere LS, Newman MG. Exposing worry’s deceit: Percentage of untrue worries in generalized anxiety disorder treatment. Behavior Therapy. 2020 May;51(3):413-23. PMID: 32402257. doi: 10.1016/j.beth.2019.07.003.

46. LaFreniere LS, Newman MG. Upregulating positive emotion in generalized anxiety disorder: A randomized controlled trial of the SkillJoy ecological momentary intervention. J Consult Clin Psychol. 2023 Jun;91(6):381-7. PMID: 36716146. doi: 10.1037/ccp0000794.

47. Sajjadian M, Uher R, Ho K, Hassel S, Milev R, Frey BN, et al. Prediction of depression treatment outcome from multimodal data: a CAN-BIND-1 report. Psychol Med. 2023 Sep;53(12):5374-84. PMID: 36004538. doi: 10.1017/S0033291722002124.

48. Liu S, Schlesinger JJ, McCoy AB, Reese TJ, Steitz B, Russo E, et al. New onset delirium prediction using machine learning and long short-term memory (LSTM) in electronic health record. J Am Med Inform Assoc. 2022 Dec 13;30(1):120-31. PMID: 36303456. doi: 10.1093/jamia/ocac210.

49. Islam R, Layek MA. StackEnsembleMind: Enhancing well-being through accurate identification of human mental states using stack-based ensemble machine learning. Informatics in Medicine Unlocked. 2023;43. doi: 10.1016/j.imu.2023.101405.

50. Varma S, Simon R. Bias in error estimation when using cross-validation for model selection. BMC Bioinformatics. 2006 Feb 23;7:Article 91. PMID: 16504092. doi: 10.1186/1471-2105-7-91.

51. Cawley GC, Talbot NLC. On over-fitting in model selection and subsequent selection bias in performance evaluation. Journal of Machine Learning Research. 2010 Jul;11(70):2079–107. PMID: WOS:000282523000006.

52. Cortes C, Vapnik V. Support-vector networks. Machine Learning. 1995 1995/09/01;20(3):273-97. doi: 10.1007/BF00994018.

53. Rothacher Y, Strobl C. Identifying informative predictor variables with random forests. Journal of Educational and Behavioral Statistics. 2023;49(4):595-629. doi: 10.3102/10769986231193327.

54. Breiman L. Random forests. Machine Learning. 2001;45(1):5-32. doi: 10.1023/A:1010933404324.

55. Guenther N, Schonlau M. Support vector machines. The Stata Journal. 2016 2016/12/01;16(4):917-37. doi: 10.1177/1536867X1601600407.

56. Hastie T, Tibshirani R, Friedman J, editors. The elements of statistical learning. New York, NY: Springer Science & Business Media; 2009.

57. Hernán MA, Robins JM. Causal inference: What if. Boca Raton: Chapman & Hall/CRC; 2023.

58. Imbens GW, Rubin DB. Causal inference for statistics, social, and biomedical sciences: An introduction. New York, NY, US: Cambridge University Press; 2015. xix, 625-xix, p. ISBN: 978-0-521-88588-1 (Hardcover).

59. Austin PC, Stuart EA. Moving towards best practice when using inverse probability of treatment weighting (IPTW) using the propensity score to estimate causal treatment effects in observational studies. Stat Med. 2015 Dec 10;34(28):3661-79. PMID: 26238958. doi: 10.1002/sim.6607.

60. VanderWeele TJ. On the distinction between interaction and effect modification. Epidemiology. 2009 Nov;20(6):863-71. PMID: 19806059. doi: 10.1097/EDE.0b013e3181ba333c.
